# Optimal Regularity and Structure of the Free Boundary for Minimizers in Cohesive Zone Models

**DOI:** 10.1007/s00205-020-01509-3

**Published:** 2020-04-10

**Authors:** L. Caffarelli, F. Cagnetti, A. Figalli

**Affiliations:** 1 Department of Mathematics, The University of Texas at Austin, 1 University Station C1200, Austin, TX 78712 USA; 2 Department of Mathematics, University of Sussex, Pevensey 2, Brighton, BN1 9QH UK; 3 Department of Mathematics, ETH Zürich, Rämistrasse 101, 8092 Zurich, Switzerland

## Abstract

We study optimal regularity and free boundary for minimizers of an energy functional arising in cohesive zone models for fracture mechanics. Under smoothness assumptions on the boundary conditions and on the fracture energy density, we show that minimizers are $C^{1, 1/2}$, and that near non-degenerate points the fracture set is $C^{1, \alpha }$, for some $\alpha \in (0, 1)$.

## Introduction

In recent years, a variational formulation of fracture evolution has been proposed by Francfort and Marigo [21], and later developed by Dal Maso and Toader [17], and Dal Maso, Francfort, and Toader [15, 16] (see also [22] and the references therein, for a variational theory of rate independent processes). Such evolution is based on the idea that at any given time the configuration of the elastic body is an absolute minimiser of the energy functional (see also [4, 11, 14, 18] in the context of plasticity where, more in general, critical points of the energy are allowed).

In this paper we study optimal regularity and free boundary for minimizers of an energy functional arising in cohesive zone models for fracture mechanics. Such models describe the situation in which the energy density of the fracture depends on the distance between the lips of the crack (see for instance [4, 11–13, 19]). We consider the energy functional associated to an elastic body occupying the open strip $\mathbb {R}^n \times (-A, A)$, with $n \ge 2$ and $A > 0$. Denoting a generic point $z \in {\mathbb {R}}^n \times (-A, A)$ by (*x*, *y*), with $x \in \mathbb {R}^n$ and $y \in (-A, A)$, we shall consider deformations ensuring that cracks can only appear on the hyperplane $\{ y = 0 \}$. The assumption of confining fractures to a given hyperplane is a standard simplification that avoids some technical difficulties but does not prevent the crack set from being irregular, thus keeping the main features of the problem.

We consider the situation in which the elastic body can only undergo deformations that are parallel to a fixed given direction lying on $\{ y = 0 \}$. In this way, the displacement can be represented by a scalar function $\text {v}: {\mathbb {R}}^n \times (-A, A) \rightarrow \mathbb {R}$. According to Barenblatt’s cohesive zone model [7], the energy associated to a displacement $\text {v} \in H^1 ({\mathbb {R}}^n \times (-A, A) {\setminus } \{ y = 0 \})$ is given by

1.1 $$\begin{aligned} E(\text {v}):=\frac{1}{2}\int _{\mathbb {R}^n \times (-A, A) {\setminus } \{ y = 0\}} | \nabla \text {v} |^2 \mathrm{d}z + \int _{\mathbb {R}^n} g (| [\text {v}] |) \, \mathrm{d}x. \end{aligned}$$

Here, $[\text {v}] = \text {v}_{\scriptscriptstyle RT} - \text {v}_{\scriptscriptstyle LT}$, where $\text {v}_{\scriptscriptstyle RT}$ and $\text {v}_{\scriptscriptstyle LT}$ are the right and left traces on $\{ y= 0 \}$ of $\text {v} \mid _{\mathbb {R}^n \times (0, A)}$ and $\text {v} \mid _{\mathbb {R}^n \times (-A, 0)}$, respectively, and $g \in C^2[0, \infty )\cap C^3 (0, \infty )$ is strictly increasing, bounded, with $g (0) = 0$ and $g'(0^+) \in (0,+\infty )$. The parameter $g' (0^+)$ has an important physical meaning, and it can be identified with the *maximal sustainable stress* of the material along $\{ y = 0\}$, see [11, Theorem 4.6]. A critical point *u* of (1.1) with boundary conditions $u_{\scriptscriptstyle A}, u_{\scriptscriptstyle -A}$ satisfies (see [11, Proposition 3.2]):

$$\begin{aligned} {\left\{ \begin{array}{ll} \Delta u = 0 \, &{}\quad \text { in } \, \mathbb {R}^{n} \times (-A, A) {\setminus } \{ y= 0\}, \\ u = u_{\scriptscriptstyle A} \, &{}\quad \text { on } \{ y = A \}, \\ u = u_{\scriptscriptstyle -A} \, &{}\quad \text { on } \{ y = - A \}, \\ \partial _{y} u_{\scriptscriptstyle RT}= \partial _{y} u_{\scriptscriptstyle LT} \, &{}\quad \text { on } \{ y= 0\}, \\ |\partial _{y} u | \le g' (0^+) \, &{}\quad \text { on } \{ y= 0\}, \\ \partial _{y} u = g' (|[u]|) \, \text {sgn} ([u]) \, &{}\quad \text { on } \{ y = 0 \} \cap \{ [u] \ne 0 \}, \end{array}\right. } \end{aligned}$$

where $\text {sgn} (\cdot )$ denotes the sign function. Note that, because $\partial _{y} u_{\scriptscriptstyle RT} = \partial _{y} u_{\scriptscriptstyle LT}$, we can use the notation $\partial _yu$ to denote the *y* derivative of *u* on $\{y=0\}$ without paying attention to the side on which the derivative is computed.

For simplicity, we will assume that $u_{\scriptscriptstyle A} (x) = - u_{\scriptscriptstyle -A}(x)$ for every $x \in \mathbb {R}^n$, and we will focus on solutions that are odd with respect to the hyperplane $\{ y = 0 \}$. In this situation, our problem reduces to the study of a function $u \in H^1 (\mathbb {R}^n \times (0,A))$ satisfying

1.2 $$\begin{aligned} {\left\{ \begin{array}{ll} \Delta u = 0 \, &{}\quad \text { in } {\mathbb {R}}^n \times (0,A), \\ u = u_{\scriptscriptstyle A} \, &{}\quad \text { on } \{ y = A \}, \\ |\partial _{y} u | \le g' (0^+) \, &{}\quad \text { on } \{ y = 0 \}, \\ \partial _{y} u = g' (2|u|) \, \text {sgn} (u) \, &{}\quad \text { on }\{ y = 0 \} \cap \{ u \ne 0 \}, \end{array}\right. } \end{aligned}$$

where we used the notation $u(x, 0) = u_{\scriptscriptstyle RT} (x, 0)$ for every $x \in \mathbb {R}^n$. In this setting, the crack $K_u$ is represented by the discontinuity set of *u*, and is given by

1.3 $$\begin{aligned} K_u := \{ (x, 0) : x \in \mathbb {R}^n, \, u (x, 0) \ne 0 \} \subset \mathbb {R}^n. \end{aligned}$$

We assume that the boundary condition $u_{\scriptscriptstyle A}$ satisfies the following:

1.4 $$\begin{aligned} u_{\scriptscriptstyle A} \in H^{1/2} ({\mathbb {R}}^n) \cap C^{2, \beta } (\mathbb {R}^n) \text { for some } \beta \in (0,1) \quad \text { and } \quad \lim _{|x| \rightarrow \infty } u_{\scriptscriptstyle A} (x) = 0.\nonumber \\ \end{aligned}$$

Under these assumptions, we want to the study optimal regularity of the restriction of *u* to $\mathbb {R}^n \times [0,A]$, and the regularity of the free boundary $\partial K_u$ (where the boundary is defined in the topology of ${\mathbb {R}}^n \times \{ 0 \}$).

A major obstacle to the regularity of solutions is the possible presence of fracture points where *u* changes sign. Indeed, at such points the normal derivative $\partial _y u (\cdot , 0)$ is discontinuous with a jump of $2 g' (0^+)$, due to the term $\text {sgn} (u)$ appearing in (1.2). Our main contribution in this paper is to show that this possibility can never occur.

Problems of this type, where two phases (in this case the sets $\{ x \in \mathbb {R}^n : u (x,0) > 0 \}$ and $\{ x \in \mathbb {R}^n : u (x,0) < 0 \}$) can “touch” at a lower dimensional free boundary, have recently been studied by Allen and Petrosyan, [3], Allen [1], and Allen, E. Lindgren and A. Petrosyan [2]. In these papers, to show the separation of phases the authors use in a clever way the Alt-Caffarelli-Friedman and the Weiss monotonicity formulas. Our approach is different and, although it requires *g* to be sufficiently smooth, it does not rely on monotonicity formulas. Therefore, it can be applied to problems where the Laplacian is replaced by more general operators.

To show the separation of phases, we begin by proving some interesting general properties of the solutions, such as the fact that the crack set $K_u$ is bounded (see Lemma 3.2 and Proposition 3.3). After that, we prove that certain regularity properties of $u_{\scriptscriptstyle A}$ “propagate” to $u (\cdot , y)$. More precisely, for every $y \in [0, A)$ we prove that $u (\cdot , y)$ is Lipschitz continuous, that $u^+ (\cdot , y):= \max \{ u (\cdot , y), 0 \}$ is semiconvex, and that $u^- (\cdot , y):= \min \{ u (\cdot , y), 0 \}$ is semiconcave, see Lemma 3.4, Lemma 3.5, and Lemma 3.6. Let us mention that, to show these regularity properties, we need to assume $2 \Vert g'' \Vert _{L^{\infty }} < 1/A$. That is, we need the size *A* of the strip to be sufficiently small, once the elastic properties of the material are given. As shown in Lemma 3.1, under this assumption critical points are unique and therefore coincide with the global minimizer. We think this bound to be sharp, and this is in agreement with an explicit example given in [11, Theorem 9.1 and Theorem 9.2], where uniqueness fails if $2 \Vert g'' \Vert _{L^{\infty }} > 1/A$.

Actually, in Lemmata 3.5 and 3.6 we prove a stronger property than the semiconvexity (resp. semiconcavity) of $u^+$ (resp. $u^-$), since we need an estimate that allows us to “connect” the behavior of $u^+$ and $u^-$ near the set $\{u=0\}$ (see Remark 3.7). This indeed plays a crucial role in the proof of Proposition 4.1, where we prove that the two phases $\{ x \in \mathbb {R}^n : u (x,0) > 0 \}$ and $\{ x \in \mathbb {R}^n : u (x,0) < 0 \}$ are well separated. We achieve this in the following way: first of all, exploiting Remark 3.7, we prove that if $(\overline{x}, 0) \in \partial K_u$ is any free boundary point where the sign of *u* changes, then $u(\cdot , 0)$ is differentiable at ${\overline{x}}$ and $\nabla _x u (\overline{x}, 0) = 0$. This, in turn, allows us to construct some suitable barriers from which we reach a contradiction.

Once we know that the sets $\{ u > 0\} \cap \{ y = 0 \}$ and $\{ u > 0 \} \cap \{ y = 0 \}$ are well separated, we can adapt to our setting the arguments used in [5, 8, 24] to prove the optimal regularity of solutions.

### Theorem 1.1

Let $u_{\scriptscriptstyle A}$ satisfy (1.4), and let $g \in C^2[0, \infty ) \cap C^3 (0, \infty )$ be strictly increasing and bounded, with $g(0)=0$ and $g'(0^+) \in (0,+\infty )$. Suppose, in addition, that $2 \Vert g'' \Vert _{L^{\infty }} < 1/A$, $\Vert g''' \Vert _{L^{\infty }} < \infty$, and that $u \in H^1 (\mathbb {R}^n \times (0,A))$ is a solution of (1.2). Then, $u \in C^{1,1/2}$.

Once both phase separation and optimal regularity of *u* are obtained, we deal with the regularity of the free boundary. To this aim, we proceed by applying more standard techniques, which are specific to operators for which monotonicity formulas are available. Assuming without loss of generality that we are at a free boundary point coming from the positive phase, we subtract from *u* the linear function $g' (0^+) y$, and then we reflect evenly with respect to the hyperplane $\{ y = 0\}$, defining

1.5 $$\begin{aligned} v (x, y):= {\left\{ \begin{array}{ll} u (x, y) - g' (0^+) y &{} \text { for every }(x, y) \in \mathbb {R}^n \times (0, A), \\ v (x, -y) &{} \text { for every } (x, y) \in \mathbb {R}^n \times (-A, 0). \end{array}\right. } \end{aligned}$$

Then, inspired by [9], we prove a variant of Almgren’s monotonicity formula. More precisely, suppose that $(0, 0) \in \partial K_u$, and set

$$\begin{aligned} \Phi _v (r):= r \frac{\mathrm{d}}{{\mathrm{d}}r} \log \left( \max \{ F_v (r) , r^{n+4} \} \right) , \qquad F_v (r) := \int _{\partial B_r} v^2 {\mathrm{d}} {\mathcal {H}}^n, \end{aligned}$$

where $B_r$ is the ball of $\mathbb {R}^{n+1}$ centred at 0 with radius *r*, and ${\mathcal {H}}^n$ denotes the Hausdorff *n*-dimensional measure. We show that there exists $C > 0$ such that for *r* sufficiently small the function $r \mapsto \Phi _v (r) e^{C r}$ is nondecreasing (see Proposition 5.1). This implies that $\Phi _v (0^+)$ exists, and we can show that either $\Phi _v (0^+) = n+3$, or $\Phi _v (0^+) \ge n+4$ (see Proposition 6.1). This allows us to classify subquadratic blow up profiles of *v*: more precisely, considering the family $\{v_r\}_{r>0}$ of functions

$$\begin{aligned} v_r (z) := \frac{v (r z)}{d_r}, \qquad \qquad d_r:= \left( \frac{F_v (r)}{r^n} \right) ^{1/2}, \end{aligned}$$

we can classify the possible limits as $r \rightarrow 0^+$ provided $\frac{d_r}{r^2}\rightarrow +\infty$.

In other words, provided *v* decays slower than quadratic, we obtain the following theorem, which is the second main result of the paper:

### Theorem 1.2

Let the assumptions of Theorem 1.1 be satisfied, and let $u \in H^1 (\mathbb {R}^n \times (0,A))$ be a solution of (1.2). Suppose that $(0,0) \in \partial K_u$, with $u (\cdot , 0) \ge 0$ near (0, 0), and let *v* be defined by (1.5). If

1.6 $$\begin{aligned} \liminf _{r \rightarrow 0^+} \frac{d_r}{r^2} = + \infty , \end{aligned}$$

then the free boundary $\partial K_u$ is of class $C^{1, \alpha }$ near (0, 0), for some $\alpha \in (0,1)$.

To prove Theorem 1.2 we show that (1.6) implies that $\Phi _v$ attains its smallest possible value, namely $\Phi _v (0^+) = n +3$, and that in this case blow up profiles of *v* are homogeneous solutions of the classical Signorini problem (that is the classical thin obstacle problem), with homogeneity degree $1/2(\Phi _v (0^+) - n)$. Thanks to this fact, the blow ups can be easily classified (see Proposition 6.2) and the result follows as in the classical theory.

The paper is organised as follows. In Section 2 we introduce the notation and the setting of the problem. We show basic regularity properties of the solution *u* in Section 3, while Section 4 is devoted to the separation of phases and the optimal regularity. Frequency formula is the subject of Section 5, and in Section 6 we study blow up profiles. Finally, in Section 7 we prove the regularity of the free boundary.

## Notation

In this brief section we introduce the notation that will be used, and we give the main assumptions. Throughout the paper, we fix $n \in {\mathbb {N}}$, with $n \ge 2$, and $A > 0$. For every point $z \in \mathbb {R}^{n} \times [-A, A]$ we will write $z = (x, y)$, with $x \in \mathbb {R}^n$ and $y \in [-A, A]$. The canonical basis of $\mathbb {R}^{n+1}$ is denoted by ${\mathbf {e}}_1,\dots , {\mathbf {e}}_{n+1}$. For $a, b \in \mathbb {R}^{n+1}$, $a \cdot b$ denotes the Euclidean scalar product between *a* and *b*, and $|\cdot |$ denotes both the absolute value in $\mathbb {R}$ and the Euclidean norm in $\mathbb {R}^n$ or $\mathbb {R}^{n+1}$, depending on the context. For every $k \in {\mathbb {N}}$, ${\mathcal {H}}^k$ stands for the Hausdorff *k*-dimensional measure. If $z = (x, y) \in \mathbb {R}^{n+1}$ and $r> 0$, we will denote by $B_r (z)$ the ball of $\mathbb {R}^{n+1}$ centered at *z* with radius *r*:

$$\begin{aligned} B_r (z) = \{ \overline{z} \in \mathbb {R}^{n+1} : | \overline{z} - z | < r \}, \end{aligned}$$

and with $B^n_r (x)$ the ball of $\mathbb {R}^{n}$ centered at *x* with radius *r*:

$$\begin{aligned} B^n_r (x) = \{ \overline{x} \in \mathbb {R}^{n} : | \overline{x} - x | < r \}. \end{aligned}$$

We will write $B_r$ and $B^n_r$ for $B^n_r (0)$ and $B^n_r (0)$, respectively, and we will use the notation ${\mathbb {S}}^{n}:= \partial B_1$ and ${\mathbb {S}}^{n-1}:= \partial B^n_1$, while $\omega _{n+1}$ denotes the $(n+1)$-dimensional Lebesgue measure of $B_1$.

Throughout all the paper, *C* will denote a universal constant, possibly different from line to line. For any function $\text {v} \in H^1 ( \mathbb {R}^n \times (-A, A) {\setminus } \{ y = 0 \})$, we will denote by $\text {v}_{\scriptscriptstyle RT}$ and $\text {v}_{\scriptscriptstyle LT}$ the right and left traces on $\{y=0\}$ of $\text {v}\mid _{\mathbb {R}^n \times (0, A)}$ and $\text {v}\mid _{\mathbb {R}^n \times (-A, 0)}$, respectively, while we set

$$\begin{aligned} \text {v}^+ := \max \{ \text {v}, 0 \} \quad \text { and } \quad \text {v}^- := \min \{ \text {v}, 0 \}, \end{aligned}$$

so that $\text {v} = \text {v}^+ + \text {v}^-$. When v is sufficiently regular, $\nabla \text {v}$ and $D^2 \text {v}$ stand for the gradient and the Hessian of $\text {v}$, while $\nabla _x \text {v}$ and $D^2_{xx} \text {v}$ are the gradient and the Hessian of the function $x \mapsto \text {v} (x, y)$. We will say that v is *homogeneous* of degree $\mu$ if v can be written as

$$\begin{aligned} \text {v} (z) = |z|^{\mu } h \left( \frac{z}{|z|} \right) , \end{aligned}$$

for some function $h : {\mathbb {S}}^{n} \rightarrow \mathbb {R}$. Let $L_0, D_0 \ge 0$. For a function $f : {\mathbb {R}}^n \rightarrow {\mathbb {R}}$, we say that *f* is Lipschitz continuous, with Lipschitz constant $L_0$, if

$$\begin{aligned} \sup _{x_1\ne x_2} \frac{|f (x_2) - f (x_1)|}{|x_2 - x_1|} \le L_0. \end{aligned}$$

Also, *f* is said to be *semiconvex*, with semiconvexity constant $D_0$, if

$$\begin{aligned} f (x + h) + f (x - h) - 2 f (x) \ge - D_0 |h|^2, \end{aligned}$$

for every $x,h \in {\mathbb {R}}^n$. Similarly, we say that *f* is *semiconcave*, with semiconcavity constant $D_0$, if

$$\begin{aligned} f (x + h) + f (x - h) - 2 f (x) \le D_0 |h|^2, \end{aligned}$$

for every $$x,h \in {\mathbb {R}}^n$$.

We are now ready to state our assumptions. In the following, $g \in C^2[0, \infty ) \cap C^3 (0, \infty )$ is strictly increasing and bounded, with $g(0)=0$ and $g'(0^+) \in (0,+\infty )$. We assume, in addition, that $2 \Vert g'' \Vert _{L^{\infty }} < 1/A$ and $\Vert g''' \Vert _{L^{\infty }} < \infty$, where $\Vert g'' \Vert _{L^{\infty }}$ and $\Vert g''' \Vert _{L^{\infty }}$ denote the $L^{\infty }$-norms of $g''$ and $g'''$, respectively. Moreover, we assume that $u_{\scriptscriptstyle A} : \mathbb {R}^n \rightarrow \mathbb {R}$ satisfies (1.4), that is

$$\begin{aligned} u_{\scriptscriptstyle A} \in H^{1/2} ({\mathbb {R}}^n) \cap C^{2, \beta } (\mathbb {R}^n) \text { for some } \beta \in (0,1) \quad \text { and } \quad \lim _{|x| \rightarrow \infty } u_{\scriptscriptstyle A} (x) = 0. \end{aligned}$$

### Remark 2.1

The assumptions above imply, in particular, that $u_{\scriptscriptstyle A}$ is Lipschitz continuous with Lipschitz constant $L_A:=\Vert \nabla u_{\scriptscriptstyle A}\Vert _{L^\infty }$. Moreover, denoting by $\lambda _{\text {min}} (x)$ and $\lambda _{\text {max}} (x)$ the smallest and largest eigenvalue of $D^2 u_{\scriptscriptstyle A} (x)$, respectively, we have that $u_{\scriptscriptstyle A}$ is semiconvex with semiconvexity constant $D_A := \Vert (\lambda _{\text {min}})^-\Vert _{L^\infty }$, and is semiconcave with semiconcavity constant $C_A := \Vert (\lambda _{\text {max}})^+\Vert _{L^\infty }$.

We will study optimal regularity and free boundary for a function $u \in H^1 (\mathbb {R}^n \times (0,A))$ solving Equation (1.2):

$$\begin{aligned} {\left\{ \begin{array}{ll} \Delta u = 0 \, &{}\quad \text { in } {\mathbb {R}}^n \times (0,A), \\ u = u_{\scriptscriptstyle A} \, &{}\quad \text { on } \{ y = A \}, \\ |\partial _{y} u | \le g' (0^+) \, &{}\quad \text { on } \{ y = 0 \}, \\ \partial _{y} u = g' (2|u|) \, \text {sgn} (u) \, &{}\quad \text { on }\{ y = 0 \} \cap \{ u \ne 0 \}. \end{array}\right. } \end{aligned}$$

Note that the equation above implies that

2.1 $$\begin{aligned} - g' (2|u (x,0)|) \le \partial _{y} u (x,0) \le g' (2|u(x,0)|) \quad \text { for every } x \in {\mathbb {R}}^n. \end{aligned}$$

Also, by the maximum principle,

$$\begin{aligned} \Vert u\Vert _{L^\infty }\le \Vert u_{\scriptscriptstyle A}\Vert _{L^\infty }<\infty . \end{aligned}$$

In the next section we prove some basic regularity properties of *u*.

## Basic Properties of the Solution

We study in this section the basic regularity properties of a solution *u* of Equation (1.2). We start by showing that condition $2 \Vert g'' \Vert _{L^{\infty }} < 1/A$ implies uniqueness.

### Lemma 3.1

Let $u_{\scriptscriptstyle A}$ satisfy (1.4), and let $g \in C^2[0, \infty )$ be strictly increasing and bounded, with $g(0)=0$ and $g'(0^+) \in (0,+\infty )$. If $2 \Vert g'' \Vert _{L^{\infty }} < 1/A$, then there exists a unique $u \in H^1 (\mathbb {R}^n \times (0,A))$ solving (1.2). In particular, there is a unique critical point of (1.1) that coincides with the global minimizer.

### Proof

Suppose, by contradiction, that there exist $u_1, u_2 \in H^1 (\mathbb {R}^n \times (0,A))$ solutions of (1.2), with $u_1 \not \equiv u_2$. In particular, since $u_1 = u_2$ on $\{ y= A \}$, this implies

3.1 $$\begin{aligned} \Vert \nabla (u_1 - u_2) \Vert ^2_{L^2 (\mathbb {R}^n \times (0, A))} > 0. \end{aligned}$$

We will prove the statement into two steps.

**Step 1:** We show that

$$\begin{aligned} \Vert \nabla (u_1 - u_2) \Vert ^2_{L^2 (\mathbb {R}^n \times (0, A))} \le 2 \Vert g'' \Vert _{L^{\infty }} \Vert u_1 - u_2 \Vert ^2_{L^2(\mathbb {R}^n)}. \end{aligned}$$

Using the weak formulation of the equation (see [11, Proposition 3.1]) we have

3.2 $$\begin{aligned}&\int _{\mathbb {R}^n \times (0, A)} \nabla u_1 \cdot \nabla \psi \, \mathrm{d}z \nonumber \\&\qquad + \int _{\mathbb {R}^n} \Big ( \psi \, g' (2 |u_1|) \, \text {sgn} (u_1) \, 1_{\{ u_1 \ne 0 \}} +\, g' (0^+) | \psi | 1_{\{ u_1 = 0 \}} \Big ) \, \mathrm{d}x \ge 0,\quad \quad \end{aligned}$$

for every $\psi \in H^1 (\mathbb {R}^n \times (0, A))$ with $\psi = 0$ on $\{ y = A \}$. Choosing $u_2 - u_1$ as test function in (3.2) we obtain

$$\begin{aligned}&\int _{\mathbb {R}^n \times (0, A)} \nabla u_1 \cdot \nabla (u_2 - u_1) \, \mathrm{d}z \\&\quad +\int _{\mathbb {R}^n} \Big ( (u_2 - u_1) \, g' (2 | u_1 |) \, \text {sgn} (u_1) \, 1_{\{ u_1 \ne 0 \}} + g' (0^+) |u_2 - u_1| 1_{\{ u_1 = 0 \}} \Big ) \, \mathrm{d}x \ge 0. \end{aligned}$$

Analogously, using the weak formulation of the equation for $u_2$, with test function $u_1 - u_2$, we get

$$\begin{aligned}&\int _{\mathbb {R}^n \times (0, A)} \nabla u_2 \cdot \nabla (u_1 - u_2) \, \mathrm{d}z \\&\quad +\int _{\mathbb {R}^n} \Big ( (u_1 - u_2) \, g' (2 | u_2 |) \, \text {sgn} (u_2) \, 1_{\{ u_2 \ne 0 \}} + g' (0^+) |(u_1 - u_2)| 1_{\{ u_2 = 0 \}} \Big ) \, \mathrm{d}x \ge 0. \end{aligned}$$

Adding together the last two relations, we obtain

$$\begin{aligned}&\Vert \nabla (u_1 - u_2) \Vert ^2_{L^2 (\mathbb {R}^n \times (0, A))} \\&\quad \le \int _{\mathbb {R}^n} \Big ( (u_2 - u_1) \, g' (2 | u_1 |) \, \text {sgn} (u_1) \, 1_{\{ u_1 \ne 0 \}} + g' (0^+) |u_2 - u_1| 1_{\{ u_1 = 0 \}} \Big ) \, \mathrm{d}x \\&\qquad + \int _{\mathbb {R}^n} \Big ( (u_1 - u_2) \, g' (2 | u_2 |) \, \text {sgn} (u_2) \, 1_{\{ u_2 \ne 0 \}} + g' (0^+) |(u_1 - u_2)| 1_{\{ u_2 = 0 \}} \Big ) \, \mathrm{d}x \\&\quad =\,\int _{\mathbb {R}^n} (u_2 - u_1) \, \Big ( g' (2 |u_1|) \, \text {sgn}(u_1) - g' (2 |u_2|) \, \text {sgn}(u_2) \Big ) \, 1_{\{ u_1 u_2 \ne 0 \}} \, \mathrm{d}x \\&\qquad +\, \int _{\mathbb {R}^n} | u_2 | \Big ( g' (0^+) - g' (2 | u_2 |) \Big ) \, 1_{\{ u_1 = 0 \} \cap \{ u_2 \ne 0 \}} \, \mathrm{d}x \\&\qquad +\, \int _{\mathbb {R}^n} | u_1 | \Big ( g' (0^+) - g' ( 2 | u_1| ) \Big ) \, 1_{\{ u_1 \ne 0 \} \cap \{ u_2 = 0 \}} \, \mathrm{d}x. \end{aligned}$$

We now observe that

$$\begin{aligned} (u_2 - u_1) \, \Big ( g' (2 |u_1|) \, \text {sgn} (u_1) - g' (2 |u_2|) \, \text {sgn}(u_2) \Big )< 0 \quad \text { whenever } u_1 u_2 <0, \end{aligned}$$

therefore,

$$\begin{aligned}&\Vert \nabla (u_1 - u_2) \Vert ^2_{L^2 (\mathbb {R}^n \times (0, A))} \\&\quad \le \int _{\mathbb {R}^n} | u_2 - u_1 | \, | \, g' (2 |u_1|) - g' (2 | u_2 |) \, | \, 1_{ \{ u_1 u_2> 0 \} } \, \mathrm{d}x \\&\qquad + \int _{\mathbb {R}^n} | u_2 | \Big ( g' (0^+) - g' (2 | u_2 |) \Big ) \, 1_{\{ u_1 = 0 \} \cap \{ u_2 \ne 0 \}} \, \mathrm{d}x \\&\qquad + \int _{\mathbb {R}^n} | u_1 | \Big ( g' (0^+) - g' (2 |u_1 |) \Big ) \, 1_{\{ u_1 \ne 0 \} \cap \{ u_2 = 0 \}} \, \mathrm{d}x \\&\quad \le 2 \int _{\mathbb {R}^n} \Vert g'' \Vert _{L^{\infty }} | u_1 - u_2|^2 1_{ \{ u_1 u_2 >0 \} } \, \mathrm{d}x \\&\qquad + 2 \int _{\mathbb {R}^n} \Vert g'' \Vert _{L^{\infty }} | u_1 - u_2|^2 1_{\{ u_1 = 0 \} \cap \{ u_2 \ne 0 \}} \, \mathrm{d}x \\&\qquad +2 \int _{\mathbb {R}^n} \Vert g'' \Vert _{L^{\infty }} | u_1 - u_2 |^2 1_{\{ u_1 \ne 0 \} \cap \{ u_2 = 0 \}} \, \mathrm{d}x \\&\quad \le 2 \Vert g'' \Vert _{L^{\infty }} \Vert u_1 - u_2 \Vert ^2_{L^2(\mathbb {R}^n)}, \end{aligned}$$

where we also used the fact that $$| u_2 - u_1 |= |\, | u_2 | - | u_1 | \,|$$ whenever $$ u_1 u_2 >0$$.

**Step 2:** We conclude.

First of all, note that

$$\begin{aligned} u_{\scriptscriptstyle A} (x) = u_i (x, A) = u_i (x, 0) + \int _0^A \partial _y u_i (x, t) \, \mathrm{d}t \qquad \text { for every }x \in \mathbb {R}^n \text { and }i = 1, 2. \end{aligned}$$

Therefore, for every $x \in \mathbb {R}^n$,

$$\begin{aligned} u_2 (x, 0) - u_1 (x, 0)&= \int _0^A \partial _y (u_1 - u_2) (x, t)\mathrm{d}t \\&\le A^{1/2} \left( \int _0^A | \nabla (u_1 - u_2) (x, t) |^2 \, \mathrm{d}t \right) ^{1/2}, \end{aligned}$$

so that

$$\begin{aligned} \Vert u_1 - u_2 \Vert ^2_{L^2(\mathbb {R}^n)} \le A \Vert \nabla (u_1 - u_2) \Vert ^2_{L^2 (\mathbb {R}^n \times (0, A))}. \end{aligned}$$

Then, thanks to Step 1

$$\begin{aligned} \Vert \nabla (u_1 - u_2) \Vert ^2_{L^2 (\mathbb {R}^n \times (0, A))}&\le 2 A \Vert g'' \Vert _{L^{\infty }} \Vert \nabla (u_1 - u_2) \Vert ^2_{L^2 (\mathbb {R}^n \times (0, A))}. \end{aligned}$$

Since $2 A \Vert g'' \Vert _{L^{\infty }} < 1$, this implies

$$\begin{aligned} \Vert \nabla (u_1 - u_2) \Vert ^2_{L^2 (\mathbb {R}^n \times (0, A))} = 0, \end{aligned}$$

against (3.1). $$\quad \square $$

We now show that $x \longmapsto u (x, 0)$ is infinitesimal as $|x| \rightarrow \infty$.

### Lemma 3.2

Let $u_{\scriptscriptstyle A}$ and *g* be as in Theorem 1.1, and let $u \in H^1 (\mathbb {R}^n \times (0,A))$ be a solution of (1.2). Then,

$$\begin{aligned} \lim _{|x| \rightarrow \infty } u (x,0) = 0. \end{aligned}$$

### Proof

Suppose, by contradiction, that there exists a sequence $\{ x_k \}_{k \in {\mathbb {N}}} \subset {\mathbb {R}}^n$ such that $|x_k| \rightarrow \infty$ and

$$\begin{aligned} \lim _{k \rightarrow \infty } u (x_k,0) = a \ne 0. \end{aligned}$$

Define now, for every $k \in {\mathbb {N}}$, the function $u_k: {\mathbb {R}}^n \times [0,A] \rightarrow {\mathbb {R}}$ as

$$\begin{aligned} u_k (x, y) : = u (x + x_k, y). \end{aligned}$$

Since $u_k$ is harmonic for every $k \in {\mathbb {N}}$ and $\{ u_k \}_{k \in {\mathbb {N}}}$ is uniformly bounded in ${\mathbb {R}}^n \times [0,A]$ and $\Vert u_k\Vert _{H^1(\mathbb {R}^n\times (0,A))}\le C$, up to subsequences we have

3.3 $$\begin{aligned} u_k \rightarrow \overline{u} \quad \text { uniformly on compact subsets of } {\mathbb {R}}^n \times [0,A] \end{aligned}$$

for some harmonic function $\overline{u}: {\mathbb {R}}^n \times [0,A] \rightarrow {\mathbb {R}}$ such that $\overline{u} (\cdot , A) \equiv 0$ and $\overline{u} (0,0) = a$, with ${\overline{u}} \in H^1 (\mathbb {R}^n \times (0,A))$. Since $u_k$ is harmonic for each *k*, we have

3.4 $$\begin{aligned} 0&= \int _{{\mathbb {R}}^n \times (0,A)} {u}_k \Delta u_k \, \mathrm{d}z = \int _{{\mathbb {R}}^n \times (0,A)} \text {div} ({u}_k \nabla u_k) \, \mathrm{d}z - \int _{{\mathbb {R}}^n \times (0,A)} |\nabla u_k|^2 \, \mathrm{d}z \nonumber \\&= \int _{{\mathbb {R}}^n} {u}_A (x+x_k) ( \partial _y u_k) (x, A) \, \mathrm{d}x - \int _{{\mathbb {R}}^n} {u}_k (x, 0) ( \partial _y u_k) (x, 0) \, \mathrm{d}x - \int _{{\mathbb {R}}^n \times (0,A)} |\nabla u_k|^2 \, \mathrm{d}z \nonumber \\&= \int _{{\mathbb {R}}^n} {u}_A (x+x_k) ( \partial _y u_k) (x, A) \, \mathrm{d}x - \int _{{\mathbb {R}}^n} |{u}_k (x, 0)|\,g'(2|{u}_k (x, 0)|) \, \mathrm{d}x - \int _{{\mathbb {R}}^n \times (0,A)} |\nabla u_k|^2 \, \mathrm{d}z\nonumber \\&\le \int _{{\mathbb {R}}^n} {u}_A (x+x_k) ( \partial _y u_k) (x, A) \, \mathrm{d}x - \int _{{\mathbb {R}}^n \times (0,A)}|\nabla u_k|^2 \, \mathrm{d}z. \end{aligned}$$

Letting $k\rightarrow \infty ,$ since $u_{\scriptscriptstyle A}(x_k+\cdot )\rightarrow 0$, we obtain

$$\begin{aligned} \int _{{\mathbb {R}}^n \times (0,A)} | \nabla \overline{u} |^2 \, \mathrm{d}z \le \liminf _{k\rightarrow \infty } \int _{{\mathbb {R}}^n \times (0,A)}|\nabla u_k|^2 \, \mathrm{d}z= 0, \end{aligned}$$

where we also used the fact that $u_k \rightharpoonup \overline{u}$ weakly in $H^1_{loc} ({\mathbb {R}}^n \times (0,A))$. Since $\overline{u}(\cdot ,A)\equiv 0$ this implies ${\overline{u}} \equiv 0$, which contradicts the fact that $\overline{u} (0,0) = a \ne 0$. $\quad \square$

We now prove that the crack set $K_u$ defined in (1.3) is bounded.

### Proposition 3.3

Let $u_{\scriptscriptstyle A}$ and *g* be as in Theorem 1.1, and let $u \in H^1 (\mathbb {R}^n \times (0,A))$ be a solution of (1.2). Then, $u (\cdot , 0)$ has compact support.

### Proof

We start by showing that there exist positive constants $R = R (g, A)$, $c = c(g, A)$, and $r = r (g, A) \in (0,1)$ with the following property: if $x_1 \in {\mathbb {R}}^n$ is such that $|x_1| > R$ and $u(x_1,0) \ne 0$, then

3.5 $$\begin{aligned} \exists \, z_1 \in \overline{B_1^n} (x_1) \quad \text { such that } \qquad \int _{B_r^n (z_1) \times (0,A)} |\nabla u|^2 \, \mathrm{d}x \, \mathrm{d}y \ge c. \end{aligned}$$

Before proving the claim, let us show that this implies the conclusion. Indeed, suppose by contradiction that the support of $u (\cdot , 0)$ is not bounded. Then, there exists a sequence $\{ x_k \}_{k \in {\mathbb {N}}} \subset {\mathbb {R}}^{n}$ with $|x_k| \rightarrow \infty$ such that $|x_k| > R$ and $u(x_k,0) \ne 0$ for every $k \in {\mathbb {N}}$. By (3.5), for every $k \in {\mathbb {N}}$ there exists $z_k \in \overline{B_1^n} (x_k)$ such that

$$\begin{aligned} \int _{B_r^n (z_k) \times (0,A)} |\nabla u|^2 \, \mathrm{d}x \, \mathrm{d}y \ge c. \end{aligned}$$

Without loss of generality, we can assume that $|x_j - x_k| \ge 4$ for every $j \ne k$, so that the balls $\{ B_r^n (z_k) \}_{k \in {\mathbb {N}}}$ are pairwise disjoint. Therefore,

$$\begin{aligned} \int _{{\mathbb {R}}^{n} \times (0,A)} |\nabla u|^2 \, \mathrm{d}x \, \mathrm{d}y \ge \sum _{k = 1}^{\infty } \int _{B^n_r (z_k) \times (0,A)} |\nabla u|^2 \, \mathrm{d}x \, \mathrm{d}y = \infty , \end{aligned}$$

against the fact that $u \in H^1 (\mathbb {R}^n \times (0, A))$.

Let us now show the claim. By Lemma 3.2,

3.6 $$\begin{aligned} \lim _{|x| \rightarrow \infty } u_{\scriptscriptstyle A} (x) = \lim _{|x| \rightarrow \infty } u(x, 0) = 0. \end{aligned}$$

Let $V : \overline{B_1^n} \times [0,A] \rightarrow {\mathbb {R}}$ be the solution of the following problem:

$$\begin{aligned} {\left\{ \begin{array}{ll} \Delta V = 0 \, &{}\quad \text { in } \, B_1^n \times (0,A), \\ V = |x|^2 \, &{}\quad \text { on } \, B_1^n \times \{ y = 0 \}, \\ V = 1 \, &{}\quad \text { on } B_1^n \times \{ y = A \}, \\ V = 1 \, &{}\quad \text { on } \partial B_1^n \times (0,A), \end{array}\right. } \end{aligned}$$

and let $a = a (g, A) > 0$ be so small that

3.7 $$\begin{aligned} \sup _{|x| \le \frac{1}{2}} | \partial _y V (x,0)|< \frac{g' (0^+)}{2 a} \qquad \text { and } \qquad g'(s) > \frac{g' (0^+)}{2} \quad \text { for } 0< s < \frac{a}{2}.\nonumber \\ \end{aligned}$$

By (3.6), there exists a constant $R=R(g, u_{\scriptscriptstyle A}) > 2$ such that

3.8 $$\begin{aligned} |u(x,0)|< \frac{a}{4} \quad \text { and } \quad |u(x,A)| = |u_{\scriptscriptstyle A}(x)| < \frac{a}{4}, \quad \text { for every } x \text { with } |x| > R - 2.\nonumber \\ \end{aligned}$$

Let $x_1 \in {\mathbb {R}}^n$ be such that $|x_1| > R$ and $u (x_1,0) > 0$ (the case $u (x_1,0) < 0$ can be treated in the same way). We will show that there exist $z_1 \in \overline{B_1^n} (x_1)$, $c > 0$, and $r \in (0, 1)$ such that (3.5) holds true.

For every $b > 0$ define $V_b (x,y) := a V (x-x_1,y) + b$ and set

$$\begin{aligned} \overline{b}:= \inf \{ b> 0 : V_b > u \text { in } B^n_1 (x_1) \times (0,A) \}. \end{aligned}$$

Note that we necessarily have $\overline{b} > 0$, since $V_0 (x_1,0) = 0 < u (x_1,0)$.

By maximum principle, there exists $(\overline{x}, \overline{y}) \in \partial (B^n_1 (x_1) \times (0,A))$ such that

$$\begin{aligned} V_{\overline{b}} (\overline{x}, \overline{y}) = u (\overline{x}, \overline{y}). \end{aligned}$$

By (3.8) it follows that $\overline{y} \ne A$, since $u(x, A)< a/4 < a+ \overline{b} = V_{\overline{b}} (x, A)$ for every *x* with $|x-x_1| \le 1$. We then have only two possibilities.

**Case i:**$\overline{y}=0$. Let us show that this is not possible. First of all, note that in this case it must be $|\overline{x}-x_1| \le 1/2$. Indeed, for every $x \in B_1^n (x_1)$ with $|x-x_1| > 1/2$

$$\begin{aligned} V_{\overline{b}} (x, 0) = a |x-x_1|^2 + \overline{b}> \frac{a}{4} > u (x, 0), \end{aligned}$$

thanks to (3.8). Thus, using (3.7) and the fact that $u (\overline{x}, 0) = V_{\overline{b}} (\overline{x}, 0) > 0$, we have

$$\begin{aligned} \frac{g' (0^+)}{2}< g' (2 u (\overline{x},0)) = \partial _y u (\overline{x},0) \le \partial _y V_{\overline{b}} (\overline{x},0) = a \, \partial _y V (\overline{x} - x_1,0) < \frac{g' (0^+)}{2}, \end{aligned}$$

which gives a contradiction.

**Case ii:**$0< \overline{y} < A$ and $|\overline{x} - x_1|=1$. Let us show that, for *a* sufficiently small, there exists a positive constant $c_1 = c_1 (g, a, A, u_{\scriptscriptstyle A})$ such that

3.9 $$\begin{aligned} 0< c_1< \overline{y}< A-c_1 < A. \end{aligned}$$

From (1.2) and (1.4) it follows that

$$\begin{aligned} | \partial _y u (x, 0)| \le g' (0^+) \qquad | \partial _y u (x, A)| \le C_0, \qquad \text { for every } x \in {\mathbb {R}}^n \end{aligned}$$

for some positive constant $C_0 > g' (0^+)$. Therefore, setting $C_1:= (C_0 - g' (0^+))/A$, by the maximum principle (note that $\partial _yu$ is harmonic)

3.10 $$\begin{aligned} - g' (0^+) - C_1 y \le \partial _y u (x,y) \le g' (0^+) + C_1 y \end{aligned}$$

for every $(x,y) \in {\mathbb {R}}^n \times [0,A]$. Therefore,

$$\begin{aligned} a< a + \overline{b}&= u (\overline{x} , \overline{y} ) = u (\overline{x} , 0 ) + \int _0^{\overline{y}} \partial _y u (\overline{x}, y) \, \mathrm{d}y \\&< \frac{a}{4} + \int _0^{\overline{y}} (g' (0^+) + C_1 y) \, \mathrm{d}y = \frac{a}{4} + g' (0^+) \overline{y} + C_1 \frac{\overline{y}^2}{2} \end{aligned}$$

where we used (3.8). The above inequality implies

$$\begin{aligned} \overline{y}> \frac{- 2 g' (0^+) + \sqrt{4 g' (0^+)^2 + 6 a C_1}}{2 C_1}> c_1>0. \end{aligned}$$

Analogously, we have

$$\begin{aligned} a < a + \overline{b}&= u (\overline{x} , \overline{y} ) = u (\overline{x} , A ) - \int _{\overline{y}}^{A} \partial _y u (\overline{x}, y) \, \mathrm{d}y \\&\le \frac{a}{4} + \int _{\overline{y}}^{A} (g' (0^+) + C_1 y) \, \mathrm{d}y = \frac{a}{4} + g' (0^+) (A - \overline{y}) + C_1 \frac{(A- \overline{y})^2}{2}, \end{aligned}$$

which implies $A - \overline{y} > c_1$, so that

$$\begin{aligned} \min \{ \overline{y} , A - \overline{y} \} > c_1, \end{aligned}$$

thus giving (3.9).

We can now show (3.5). At the contact point, we have

$$\begin{aligned} u (\overline{x} , \overline{y} ) = V_{\overline{b}} (\overline{x} , \overline{y} ) = a + \overline{b}. \end{aligned}$$

Then, by Harnack inequality and by (3.9), there exists a radius $r = r (c_1) \in (0, 1)$ such that

$$\begin{aligned} u(x,y) \ge \frac{a}{2} \qquad \text { for every } (x,y) \in B^n_r (\overline{x}) \times (\overline{y} - r , \overline{y} + r). \end{aligned}$$

The inequality above implies that for every $x \in B^n_r (\overline{x})$ (note that $|x| > R - 2$ for $x \in B^n_r (\overline{x})$, so we can use (3.8))

$$\begin{aligned} \frac{a}{4} \le u(x,\overline{y}) - u(x,A) \le \int _{{\bar{y}}}^A | \partial _y u |( x,y)\,\mathrm{d}y \le \sqrt{A} \left( \int _0^A (\partial _y u)^2(x,y)\,\mathrm{d}y \right) ^{\frac{1}{2}}, \end{aligned}$$

from which

$$\begin{aligned} \int _0^A |\nabla u|^2(x,y)\,\mathrm{d}y \ge \frac{a^2}{16 A} \qquad \qquad \forall \, x \in B^n_r (\overline{x}). \end{aligned}$$

Integrating with respect to *x*, we obtain

$$\begin{aligned} \int _{B^n_r(\overline{x}) \times (0,A)} |\nabla u|^2 \, \mathrm{d}x \, \mathrm{d}y \ge \frac{a^2 {\mathcal {H}}^n (B^n_r(\overline{x}))}{16 A }. \end{aligned}$$

Setting

$$\begin{aligned} z_1:= \overline{x}, \qquad \text { and }\qquad c := \frac{a^2 {\mathcal {H}}^n (B^n_r(\overline{x}))}{16 A}, \end{aligned}$$

the claim follows. $\quad \square$

We now show that, under the assumption $2A\Vert g '' \Vert _{L^{\infty }}<1,$ the Lipschitz continuity of $u_{\scriptscriptstyle A}$ implies the Lipschitz continuity of $u (\cdot , y)$, uniformly with respect to *y*.

### Lemma 3.4

Let $u_{\scriptscriptstyle A}$ and *g* be as in Theorem 1.1, let $u \in H^1 (\mathbb {R}^n \times (0,A))$ be a solution of (1.2), and let $L_A$ be given by Remark 2.1. Then, for every $y \in [0,A]$ the function $u (\cdot , y)$ is Lipschitz continuous, with Lipschitz constant $\frac{L_A}{1-2A\Vert g '' \Vert _{L^{\infty }}}$.

### Proof

Let $h \in {\mathbb {R}}^n {\setminus } \{ 0 \}, \alpha > 0$, and define for every $C > 0$

$$\begin{aligned} u^{h, \alpha }_C (x,y) : = u (x+h,y) + C |h| \left[ 1 + \alpha \left( 1 - \frac{y}{A} \right) \right] , \qquad (x,y) \in \mathbb {R}^n \times [0,A]. \end{aligned}$$

Setting $C^{\alpha }_h: = \inf \left\{ C> 0 : u^{h, \alpha }_C > u \right\}$, we claim that

3.11 $$\begin{aligned} C^{\alpha }_h \le L_A, \qquad \text { for } \alpha >\frac{2A\Vert g '' \Vert _{L^{\infty }}}{1- 2A\Vert g '' \Vert _{L^{\infty }}}. \end{aligned}$$

Let us first show that the claim proves the lemma. Indeed, if (3.11) is true then for every $(x,y) \in {\mathbb {R}}^n \times [0,A]$ we have

$$\begin{aligned} u (x+h,y) + L_A(1+\alpha ) |h|&\ge u (x+h,y) + L_A |h| \left[ 1 + \alpha \left( 1 - \frac{y}{A} \right) \right] \\&\ge u (x+h,y) + C^{\alpha }_h |h| \left[ 1 + \alpha \left( 1 - \frac{y}{A} \right) \right] \ge u (x, y). \end{aligned}$$

Since *x*, *y* and *h* are arbitrary, from the last inequality and letting $\alpha \rightarrow \frac{2A\Vert g '' \Vert _{L^{\infty }}}{1- 2A\Vert g '' \Vert _{L^{\infty }}}$, we get

$$\begin{aligned} |u (x+h,y) - u (x,y)| \le \frac{L_A}{1-2A\Vert g '' \Vert _{L^{\infty }}} |h| , \end{aligned}$$

thus concluding.

Let us now prove the claim. By maximum principle and thanks to (3.6), there exists $(\overline{x}, \overline{y}) \in \mathbb {R}^n \times \{ 0, A\}$ such that

$$\begin{aligned} 0 = u^{h, \alpha }_{C^{\alpha }_h} (\overline{x}, \overline{y}) - u (\overline{x}, \overline{y}) = \inf _{\mathbb {R}^n \times [0,A]} ( u^{h, \alpha }_{C^{\alpha }_h} - u ). \end{aligned}$$

In the following we assume $C^{\alpha }_h > 0$, since otherwise (3.11) is trivially satisfied. We have two possibilities.

**Case 1:**$\overline{y} = A$. Since $u_{\scriptscriptstyle A} (\cdot )$ is Lipschitz continuous, at the contact point $(\overline{x}, A)$ we have

$$\begin{aligned} - L_A |h| \le u_{\scriptscriptstyle A} (\overline{x}+h) - u_{\scriptscriptstyle A} (\overline{x}) = - C^{\alpha }_h |h| , \end{aligned}$$

from which (3.11) follows.

**Case 2:**$\overline{y} =0$. We conclude the proof of the lemma, showing that for $\alpha$ sufficiently large this case is impossible. At the contact point, the following equality holds true:

3.12 $$\begin{aligned} u (\overline{x}+h,0) + (1+ \alpha ) C^{\alpha }_h |h| = u^{h, \alpha }_{C^{\alpha }_h} (\overline{x},0) = u (\overline{x},0). \end{aligned}$$

We consider now three possible subcases, in which we will always reach a contradiction.

**Case 2a:**$\overline{y} =0$**and**$u (\overline{x},0) \le 0$. Thanks to (3.12), it has to be $u (\overline{x}+h,0) \le - (1 + \alpha ) C^{\alpha }_h |h| < 0$. Therefore, recalling (2.1) we get

$$\begin{aligned}&- g' (- 2 u (\overline{x},0 ) ) \le \partial _y u (\overline{x},0 ) \le \partial _y u^{h, \alpha }_{C^{\alpha }_h} (\overline{x},0) \\&\quad = \partial _y u (\overline{x} + h,0) - \frac{\alpha C^{\alpha }_h |h|}{A} \\&\quad = - g' ( - 2 u (\overline{x} + h,0) ) - \frac{\alpha C^{\alpha }_h |h|}{A} \\&\quad = - g' \big (- 2 u (\overline{x},0 ) + 2 (1+ \alpha ) C^{\alpha }_h |h| \big ) - \frac{\alpha C^{\alpha }_h |h|}{A} \\&\quad \le - g' (- 2 u (\overline{x},0 ) ) + C^{\alpha }_h |h| \left( 2 (1+ \alpha ) \Vert g '' \Vert _{L^{\infty }} - \frac{\alpha }{A} \right) \\&\quad = - g' (- 2 u (\overline{x},0 ) ) + C^{\alpha }_h |h| \left[ 2\Vert g '' \Vert _{L^{\infty }} + \alpha \left( 2\Vert g '' \Vert _{L^{\infty }} - \frac{1}{A} \right) \right] \\&\quad < - g' (- 2 u (\overline{x},0 ) ), \end{aligned}$$

for $$\alpha >\frac{2A\Vert g '' \Vert _{L^{\infty }}}{1- 2A\Vert g '' \Vert _{L^{\infty }}}$$.

**Case 2b:**$\overline{y} =0$**with**$u (\overline{x},0) > 0$**and**$u (\overline{x}+h,0) < 0$. In this case we have

$$\begin{aligned}&0< g' (2 u (\overline{x},0 ) ) = \partial _y u (\overline{x},0 ) \le \partial _y u^{h, \alpha }_{C^{\alpha }_h} (\overline{x},0) = \partial _y u (\overline{x} + h,0) - \frac{\alpha C^{\alpha }_h |h|}{A} \\&= - g' ( - 2 u (\overline{x} + h,0) ) - \frac{\alpha C^{\alpha }_h |h|}{A} < 0, \end{aligned}$$

which is still impossible.

**Case 2c:**$\overline{y} =0$**with**$u (\overline{x},0) > 0$**and**$u (\overline{x}+h,0) \ge 0$. This follows as in case 2a:

$$\begin{aligned}&g' (2 u (\overline{x},0 ) ) = \partial _y u (\overline{x},0 ) \le \partial _y u^{h, \alpha }_{C^{\alpha }_h} (\overline{x},0) \\&\quad = \partial _y u (\overline{x} + h,0) - \frac{\alpha C^{\alpha }_h |h|}{A} \le g' ( 2 u (\overline{x} + h,0) ) - \frac{\alpha C^{\alpha }_h |h|}{A} \\&\quad = g' \big (2 u (\overline{x},0 ) - 2 (1+ \alpha ) C^{\alpha }_h |h| \big ) - \frac{\alpha C^{\alpha }_h |h|}{A} \\&\quad \le g' (2 u (\overline{x},0 ) ) + C^{\alpha }_h |h| \left( 2 (1+ \alpha ) \Vert g '' \Vert _{L^{\infty }} - \frac{\alpha }{A} \right) \\&\quad = g' (2 u (\overline{x},0 ) ) + C^{\alpha }_h |h| \left[ 2\Vert g '' \Vert _{L^{\infty }} + \alpha \left( 2\Vert g '' \Vert _{L^{\infty }} - \frac{1}{A} \right) \right] \\&\quad < g' (2 u (\overline{x},0 ) ), \end{aligned}$$

for $\alpha >\frac{2A\Vert g '' \Vert _{L^{\infty }}}{1- 2A\Vert g '' \Vert _{L^{\infty }}}$. This proves the claim and, in turn, the lemma. $\quad \square$

We now show a property that implies the semiconvexity of $u^+ (\cdot , y)$, for any $y \in [0,A]$.

### Lemma 3.5

Let $u_{\scriptscriptstyle A}$ and *g* be as in Theorem 1.1, and let $u \in H^1 (\mathbb {R}^n \times (0,A))$ be a solution of (1.2). Then, there exists $\overline{D} > 0$ such that for every $(x, y) \in \mathbb {R}^n \times [0,A]$,

$$\begin{aligned} \left[ u(x+h,y) + u(x-h,y) + \overline{D} |h|^2 \right] ^+ \ge 2 u^+ (x, y) \qquad \forall \, h \in \mathbb {R}^n. \end{aligned}$$

In particular, for every $y \in [0,A]$ the function $u^+ (\cdot , y)$ is semiconvex, with semiconvexity constant $\overline{D}$.

An analogous result holds true for $u^-$.

### Lemma 3.6

Let $u_{\scriptscriptstyle A}$ and *g* be as in Theorem 1.1, and let $u \in H^1 (\mathbb {R}^n \times (0,A))$ be a solution of (1.2). Then, there exists $\overline{C} > 0$ such that for every $(x, y) \in \mathbb {R}^n \times [0,A]$,

$$\begin{aligned} \left[ u(x+h,y) + u(x-h,y) - \overline{C} |h|^2 \right] ^- \le 2 u^- (x, y) \qquad \forall \, h \in \mathbb {R}^n. \end{aligned}$$

In particular, for every $y \in [0,A]$ the function $u^- (\cdot , y)$ is semiconcave, with semiconcavity constant $\overline{C}$.

The following remark will be useful in the proof of Proposition 4.1.

### Remark 3.7

Combining Lemmata 3.5 and 3.6 , we obtain

$$\begin{aligned} \left[ u(x+h,y) + u(x-h,y) + \overline{D} |h|^2 \right] ^+ \ge 2 u^+ (x, y) \ge 2 u (x, y) \\ \ge 2 u^- (x, y) \ge \left[ u(x+h,y) + u(x-h,y) - \overline{C} |h|^2 \right] ^- , \end{aligned}$$

for every $(x, y) \in \mathbb {R}^n \times [0,A]$, and $h \in \mathbb {R}^n$.

### Remark 3.8

Let $L_A$, $D_A$ and $C_A$ be given by Remark 2.1. A careful inspection of the proof of Lemma 3.5 shows that one can choose

$$\begin{aligned} \overline{D} = \frac{1}{c_A} \left[ D_A + \frac{4 B_{A,g}}{c_A^2} \right] \quad \text { and } \quad \overline{C} = \frac{1}{c_A} \left[ C_A + \frac{4 B_{A,g}}{c_A^2} \right] , \end{aligned}$$

where

$$\begin{aligned} c_A:= 1 - 2 A \Vert g '' \Vert _{L^{\infty }}, \qquad B_{A,g}:= A L_A \max \{ L_A \Vert g''' \Vert _{L^{\infty }} , 2 c_A c_g \Vert g'' \Vert _{L^{\infty }} \}, \end{aligned}$$

and $c_g > 0$ is a positive constant such that

3.13 $$\begin{aligned} 4 \frac{L_A}{c_A} \Vert g'' \Vert _{L^{\infty }} t + D_A \Vert g'' \Vert _{L^{\infty }} t^2 < g' (0^+) \quad \text { for every } t \in [0, 1/c_g). \end{aligned}$$

We only give the proof of Lemma 3.5, since that one of Lemma 3.6 is analogous.

### Proof of Lemma 3.5

For every $h \in {\mathbb {R}}^n {\setminus } \{ 0 \}$, $\alpha >0$, $\varepsilon > 0$, and $C > 0$, we define the function

$$\begin{aligned} u^{h, \alpha , \varepsilon }_C (x,y) : = \left[ \frac{u(x+h,y) + u(x-h,y) + C|h|^2}{2} + \alpha \, C|h|^2 \left( 1 - \frac{y}{A} \right) \right] ^+ + \varepsilon | h|^2, \end{aligned}$$

and set $C^{\alpha , \varepsilon }_h:= \inf \{ C> 0 : u^{h, \alpha , \varepsilon }_C > u^+ \text { in }\mathbb {R}^n \times [0,A]\}$. We claim that

3.14 $$\begin{aligned} C^{\alpha , \varepsilon }_h \le \max \{D_A - 2 \varepsilon , f_{\varepsilon } (\alpha ) \} \quad \text { for every } \, \, \alpha > \frac{A\Vert g '' \Vert _{L^{\infty }}}{c_A} \, \, \text { and } \, \, 0< \varepsilon < D_A/2,\nonumber \\ \end{aligned}$$

where

$$\begin{aligned} f_{\varepsilon } (\alpha ) := 2 \frac{B_{A,g} + \varepsilon c_A^2 A \Vert g'' \Vert _{L^{\infty }}}{ c_A^2 (\alpha c_A - A \Vert g'' \Vert _{L^{\infty }} )}, \end{aligned}$$

and the constants $c_A$, $B_{A, g}$, and $c_g$ are defined in Remark 3.8. Before proving the claim, let us show how this will imply the lemma.

Setting

$$\begin{aligned} G_{\varepsilon } (\alpha ):= (1 + 2 \alpha ) \max \{D_A - 2 \varepsilon , f_{\varepsilon } (\alpha ) \}, \end{aligned}$$

from (3.14) and by definition of $C^{\alpha , \varepsilon }_h$ it follows that

3.15 $$\begin{aligned}&\left[ u(x+h,y) + u(x-h,y) + G_{\varepsilon } (\alpha ) |h|^2 \right] ^+ + 2 \varepsilon | h|^2 \nonumber \\&\quad \ge \left[ u(x+h,y) + u(x-h,y) + C^{\alpha , \varepsilon }_h (1 + 2 \alpha ) |h|^2 \right] ^+ + 2 \varepsilon | h|^2 \nonumber \\&\quad \ge \left[ u(x+h,y) + u(x-h,y) + C^{\alpha , \varepsilon }_h \left( 1 + 2 \alpha \left( 1 - \frac{y}{A} \right) \right) |h|^2 \right] ^+ + 2 \varepsilon | h|^2 \nonumber \\&\quad \ge 2 u^+ (x, y), \end{aligned}$$

for every $(x, y) \in \mathbb {R}^n \times [0, A]$, $\alpha > A\Vert g '' \Vert _{L^{\infty }} / c_A$, and $\varepsilon \in (0, D_A/2)$. One can check that for every fixed $\varepsilon \in (0, D_A/2)$

$$\begin{aligned} G_{\varepsilon } (\alpha ) = {\left\{ \begin{array}{ll} (1 + 2 \alpha ) f_{\varepsilon } (\alpha ) &{}\text { for } A\Vert g '' \Vert _{L^{\infty }} / c_A< \alpha < \alpha _{\varepsilon }, \\ (1 + 2 \alpha ) (D_A - 2 \varepsilon ) &{}\text { for } \alpha \ge \alpha _{\varepsilon }, \end{array}\right. } \end{aligned}$$

where

$$\begin{aligned} \alpha _{\varepsilon }:= \frac{A \Vert g'' \Vert _{L^{\infty }}}{c_A} + 2 \frac{B_{A,g} + \varepsilon c_A^2 A \Vert g'' \Vert _{L^{\infty }}}{c_A^3 (D_A - 2 \varepsilon )}. \end{aligned}$$

From this, it follows that for every $\varepsilon \in (0, D_A/2)$

$$\begin{aligned}&\min \left\{ G_{\varepsilon } (\alpha ) : \alpha > A\Vert g '' \Vert _{L^{\infty }} / c_A \right\} = G_{\varepsilon } (\alpha _{\varepsilon }) = \overline{D} - 2 \varepsilon , \end{aligned}$$

with $\overline{D}$ defined in Remark 3.8. Therefore, minimizing in $\alpha$ the left hand side of (3.15) we obtain

$$\begin{aligned} \left[ u(x+h,y) + u(x-h,y) + ( \overline{D} - 2 \varepsilon ) |h|^2 \right] ^+ + 2 \varepsilon | h|^2 \ge 2 u^+ (x, y), \end{aligned}$$

for every $(x, y) \in \mathbb {R}^n \times [0, A]$, and $\varepsilon \in (0, D_A/2)$. Taking the limit as $\varepsilon \rightarrow 0^+$ we conclude.

Let us now show (3.14). By definition of $C^{\alpha , \varepsilon }_h$, the maximum principle, and thanks to (3.6), there exists $(\overline{x}, \overline{y}) \in \mathbb {R}^n \times \{ 0, A \}$ such that

3.16 $$\begin{aligned} 0 = u^{h, \alpha }_{C^{\alpha , \varepsilon }_h} (\overline{x}, \overline{y}) - u^+ (\overline{x}, \overline{y}) = \inf _{{\overline{\Omega }}^+} ( u^{h, \alpha }_{C^{\alpha , \varepsilon }_h} - u^+ ). \end{aligned}$$

In what follows we assume that $C^{\alpha , \varepsilon }_h > 0$, since otherwise (3.14) is trivially satisfied. We have two possibilities.

**Case 1:**$\overline{y} = A$. At the contact point $(\overline{x}, A)$ we have

$$\begin{aligned} u^+_A (\overline{x}) = \left[ \frac{ u_{\scriptscriptstyle A} (\overline{x}+h) + u_{\scriptscriptstyle A} (\overline{x}-h)+ C^{\alpha , \varepsilon }_h |h|^2}{2} \right] ^+ + \varepsilon |h|^2 >0, \end{aligned}$$

so that $u^+_A (\overline{x}) = u_{\scriptscriptstyle A} (\overline{x}) > 0$. Therefore

$$\begin{aligned} u_{\scriptscriptstyle A} (\overline{x})&= \left[ \frac{ u_{\scriptscriptstyle A} (\overline{x}+h) + u_{\scriptscriptstyle A} (\overline{x}-h)+ C^{\alpha , \varepsilon }_h |h|^2}{2} \right] ^+ + \varepsilon |h|^2 \\&\ge \frac{ u_{\scriptscriptstyle A} (\overline{x}+h) + u_{\scriptscriptstyle A} (\overline{x}-h)+ C^{\alpha , \varepsilon }_h |h|^2}{2} + \varepsilon |h|^2 \\&\ge \frac{ 2 u_{\scriptscriptstyle A} (\overline{x}) - D_A |h|^2 + C^{\alpha , \varepsilon }_h |h|^2}{2} + \varepsilon |h|^2 = u_{\scriptscriptstyle A} (\overline{x}) + \frac{1}{2} \left( C^{\alpha , \varepsilon }_h - D_A + 2 \varepsilon \right) |h|^2, \end{aligned}$$

which implies

3.17 $$\begin{aligned} C^{\alpha , \varepsilon }_h \le D_A - 2 \varepsilon . \end{aligned}$$

**Case 2:**$\overline{y} =0$. At the contact point $(\overline{x},0)$ we have

3.18 $$\begin{aligned} 0< \left[ u(\overline{x} +h,0) + u(\overline{x} -h,0) + C^{\alpha , \varepsilon }_h (1 + 2 \alpha )|h|^2 \right] ^+ + 2 \varepsilon | h|^2 = 2 u^+ (\overline{x}, 0).\nonumber \\ \end{aligned}$$

Therefore, $$u^+ (\overline{x}, 0) = u (\overline{x}, 0)$$ and

3.19 $$\begin{aligned} 0&< g' (2 u (\overline{x}, 0)) = \partial _y u (\overline{x}, 0) \le (\partial _y u^{h, \alpha }_{C^{\alpha , \varepsilon }_h}) (\overline{x},0) \nonumber \\&= \partial _y \left\{ \left[ \frac{u(\overline{x}+h,y) + u(\overline{x}-h,y) + C^{\alpha , \varepsilon }_h |h|^2}{2} + \alpha \, C^{\alpha , \varepsilon }_h |h|^2 \left( 1 - \frac{y}{A} \right) \right] ^+ \right\} \mid _{y = 0}. \end{aligned}$$

From the fact that the right hand side in the above expression is positive, it follows that

$$\begin{aligned} \frac{u(\overline{x}+h,y) + u(\overline{x}-h,y) + C^{\alpha , \varepsilon }_h |h|^2}{2} + \alpha \, C^{\alpha , \varepsilon }_h |h|^2 \left( 1 - \frac{y}{A} \right) \ge 0 \quad \text { for}~y~\text {close to}~ {0}, \end{aligned}$$

and from (3.19) we get

3.20 $$\begin{aligned} 0 <&g' (2 u (\overline{x}, 0)) \le \frac{1}{2} \left[ \partial _y u (\overline{x} + h,0) + \partial _y u (\overline{x} - h,0) \right] - \frac{\alpha C^{\alpha , \varepsilon }_h |h|^2}{A}. \end{aligned}$$

Moreover, identity (3.18) becomes

3.21 $$\begin{aligned} u(\overline{x} +h,0) + u(\overline{x} -h,0) + C^{\alpha , \varepsilon }_h (1 + 2 \alpha )|h|^2 + 2 \varepsilon | h|^2 = 2 u (\overline{x}, 0),\qquad \end{aligned}$$

Observing now that the role played by $u (\overline{x} + h,0)$ and $u (\overline{x} - h,0)$ is symmetric, we only need to consider three subcases.

**Case 2a:**$\overline{y} =0$**with**$u (\overline{x} + h,0) \ge 0$**and**$u (\overline{x} - h,0) \ge 0$. In this case, recalling (2.1), from relation (3.20) we obtain

3.22 $$\begin{aligned} 0 <&g' (2 u (\overline{x}, 0)) \le \frac{1}{2} \left[ g' (2 u (\overline{x} + h,0) ) + g' (2 u (\overline{x} - h,0) ) \right] - \frac{\alpha C^{\alpha , \varepsilon }_h |h|^2}{A}. \end{aligned}$$

Let us now show that for every $a,b \ge 0$

3.23 $$\begin{aligned} \frac{g' (a) + g' (b)}{2} \le g' \left( \frac{a+b}{2} \right) + \frac{1}{8} \Vert g''' \Vert _{L^{\infty }} ( a-b )^2. \end{aligned}$$

Indeed, there exist $\theta , \tau \in (0,1)$ such that

$$\begin{aligned} g' (a)&= g' \left( \frac{a+b}{2} + \frac{a-b}{2} \right) = g' \left( \frac{a+b}{2} \right) + g'' \left( \frac{a+b}{2} \right) \frac{a-b}{2} \\&\quad + \frac{1}{2} g''' \mid _{ \frac{a+b}{2} + \theta \frac{a-b}{2}} \left( \frac{a-b}{2} \right) ^2, \end{aligned}$$

and

$$\begin{aligned} g' (b)&= g' \left( \frac{a+b}{2} - \frac{a-b}{2} \right) = g' \left( \frac{a+b}{2} \right) - g'' \left( \frac{a+b}{2} \right) \frac{a-b}{2} \\&\quad + \frac{1}{2} g''' \mid _{\frac{a+b}{2} - \tau \frac{a-b}{2}} \left( \frac{a-b}{2} \right) ^2. \end{aligned}$$

Summing up the last two relations we obtain the claim. Applying (3.23) with $a = 2 u (\overline{x}+h,0)$ and $b = 2 u (\overline{x}-h,0)$, and using (3.21), relation (3.22) gives

$$\begin{aligned} g' (2 u (\overline{x}, 0))&\le g' \big (2 u (\overline{x},0) - (1 + 2 \alpha ) C^{\alpha , \varepsilon }_h |h|^2 - 2 \varepsilon |h|^2 \big ) \\&\quad + \frac{1}{2} \Vert g''' \Vert _{L^{\infty }} ( u (\overline{x}+h,0) - u (\overline{x}-h,0) )^2 - \frac{\alpha C^{\alpha , \varepsilon }_h |h|^2}{A}. \end{aligned}$$

By Lemma 3.4 it follows that $u (\cdot , 0)$ is Lipschitz continuous, with Lipschitz constant $L_A/c_A$. Therefore, recalling that $c_A=1-2A\Vert g'' \Vert _{L^{\infty }},$ we get

$$\begin{aligned}&g' (2 u (\overline{x}, 0)) \le g' (2 u (\overline{x},0))\\&\qquad + \big [(1 + 2 \alpha ) C^{\alpha , \varepsilon }_h + 2 \varepsilon \big ] |h|^2 \Vert g'' \Vert _{L^{\infty }} + 2 \frac{L^2_A}{c_A^2} \Vert g''' \Vert _{L^{\infty }} |h|^2 - \frac{\alpha C^{\alpha , \varepsilon }_h |h|^2}{A} \\&\quad = g' (2 u (\overline{x},0)) + |h|^2 \left[ 2 \frac{L^2_A}{c_A^2} \Vert g''' \Vert _{L^{\infty }} \right. \\&\qquad \left. + 2 \varepsilon \Vert g'' \Vert _{L^{\infty }} + C^{\alpha , \varepsilon }_h\left( \Vert g'' \Vert _{L^{\infty }} - \alpha \frac{c_A}{A} \right) \right] \\&\quad = g' (2 u (\overline{x},0)) + |h|^2 \left[ 2 \frac{L^2_A}{c_A^2} \Vert g''' \Vert _{L^{\infty }} + 2 \varepsilon \Vert g'' \Vert _{L^{\infty }} - C^{\alpha , \varepsilon }_h \frac{\alpha c_A - A \Vert g'' \Vert _{L^{\infty }}}{A} \right] . \end{aligned}$$

The inequality above is only possible if the last term in the right hand side is non-negative, that is, if

3.24 $$\begin{aligned} C^{\alpha , \varepsilon }_h \le \frac{2 A ( L^2_A \Vert g''' \Vert _{L^{\infty }} + \varepsilon c_A^2 \Vert g'' \Vert _{L^{\infty }} )}{ c_A^2 (\alpha c_A - A \Vert g'' \Vert _{L^{\infty }} )}. \end{aligned}$$

**Case 2b:**$\overline{y} =0$**with**$u (\overline{x} + h,0) \ge 0$**and**$u (\overline{x} - h,0) < 0$. In this case, recalling (2.1), (3.20) implies that

3.25 $$\begin{aligned} 0 < g' (2 u (\overline{x}, 0)) \le \frac{1}{2} \left[ g' (2 u (\overline{x} + h,0) ) - g' (2 |u (\overline{x} - h,0)| ) \right] - \frac{\alpha C^{\alpha , \varepsilon }_h |h|^2}{A}. \end{aligned}$$

By Lemma 3.4, $u (\cdot , 0)$ is Lipschitz continuous, with Lipschitz constant $L_A/c_A$. Therefore, the right hand side of the above expression can be estimated as follows:

$$\begin{aligned}&\frac{1}{2} \left[ g' (2 | u (\overline{x} + h,0)| ) - g' (2 |u (\overline{x} - h,0)| ) \right] - \frac{\alpha C^{\alpha , \varepsilon }_h |h|^2}{A} \\&\le \Vert g'' \Vert _{L^{\infty }} \big | \, |u (\overline{x} - h,0)| - | u (\overline{x} + h,0) | \, \big | - \frac{\alpha C^{\alpha , \varepsilon }_h |h|^2}{A} \\&\le 2 \frac{L_A}{c_A} \Vert g'' \Vert _{L^{\infty }} | h | - \frac{\alpha C^{\alpha , \varepsilon }_h |h|^2}{A}. \end{aligned}$$

On the other hand, thanks to (3.21) we can estimate the left hand side of (3.25) as

$$\begin{aligned}&g' (2 u (\overline{x}, 0)) = g' \big ( | u(\overline{x} +h,0)| - | u(\overline{x} -h,0)| + C^{\alpha , \varepsilon }_h (1 + 2 \alpha )|h|^2 + 2 \varepsilon | h|^2 \big ) \\&\ge g' (0^+) - \Vert g'' \Vert _{L^{\infty }} \big | | u(\overline{x} +h,0)| - | u(\overline{x} -h,0)| \big | - \Vert g'' \Vert _{L^{\infty }} [ C^{\alpha , \varepsilon }_h (1 + 2 \alpha ) + 2 \varepsilon ] |h|^2 \\&\ge g' (0^+) - 2 \frac{L_A}{c_A} \Vert g'' \Vert _{L^{\infty }} | h | - \Vert g'' \Vert _{L^{\infty }} [ C^{\alpha , \varepsilon }_h (1 + 2 \alpha ) + 2 \varepsilon ] |h|^2. \end{aligned}$$

Combining the last two inequalities and (3.25) we obtain

3.26 $$\begin{aligned} g' (0^+)&\le 4 \frac{L_A}{c_A} \Vert g'' \Vert _{L^{\infty }} | h | + \Vert g'' \Vert _{L^{\infty }} [ C^{\alpha , \varepsilon }_h (1 + 2 \alpha ) + 2 \varepsilon ]|h|^2 - \frac{\alpha C^{\alpha , \varepsilon }_h |h|^2}{A} \nonumber \\&= 4 \frac{L_A}{c_A} \Vert g'' \Vert _{L^{\infty }} | h | + \left[ 2 \varepsilon \Vert g'' \Vert _{L^{\infty }} - C^{\alpha , \varepsilon }_h \frac{\alpha c_A - A \Vert g'' \Vert _{L^{\infty }}}{A} \right] |h|^2. \end{aligned}$$

We now distinguish two subcases.

**Case 2bi: Small values of** |*h*|. Let $c_g > 0$ be defined by (3.13). From (3.26) it follows that for every $| h | \in [0, 1/c_g)$ we have

$$\begin{aligned} g' (0^+)&\le 4 \frac{L_A}{c_A} \Vert g'' \Vert _{L^{\infty }} | h | + 2 \varepsilon \Vert g'' \Vert _{L^{\infty }} |h|^2 \\&< 4 \frac{L_A}{c_A} \Vert g'' \Vert _{L^{\infty }} | h | + D_A \Vert g'' \Vert _{L^{\infty }} |h|^2 < g' (0^+), \end{aligned}$$

which is impossible. Therefore, (3.26) can only be satisfied for $| h | \ge 1/c_g$.

**Case 2bii:** |*h*| **large.** Suppose now that $| h | \ge 1/c_g$, where $c_g$ is given by (3.13). Then, $|h| \le c_g |h|^2$ and thanks to (3.26) we obtain

$$\begin{aligned} g' (0^+)&\le \left[ \left( 4 \frac{L_A c_g}{ c_A} + 2 \varepsilon \right) \Vert g'' \Vert _{L^{\infty }} - C^{\alpha , \varepsilon }_h \frac{\alpha c_A - A \Vert g'' \Vert _{L^{\infty }}}{A} \right] |h|^2. \end{aligned}$$

Last inequality is impossible, unless

3.27 $$\begin{aligned} C^{\alpha , \varepsilon }_h< & {} \frac{\left( 4 \frac{L_A c_g}{c_A} + 2 \varepsilon \right) A \Vert g'' \Vert _{L^{\infty }}}{\alpha c_A - A \Vert g'' \Vert _{L^{\infty }}} \nonumber \\= & {} \frac{2 A ( 2 L_A c_A c_g \Vert g'' \Vert _{L^{\infty }} + \varepsilon c_A^2 \Vert g'' \Vert _{L^{\infty }} )}{ c_A^2 (\alpha c_A - A \Vert g'' \Vert _{L^{\infty }} )}. \end{aligned}$$

**Case 2c:**$\overline{y} =0$**with**$u (\overline{x} + h,0) < 0$**and**$u (\overline{x} - h,0) < 0$. In this case, inequality (3.20) becomes

$$\begin{aligned} 0<&g' (2 u (\overline{x}, 0)) \le - \frac{1}{2} \left[ g' (|u (\overline{x} + h,0)|) + g' (|u (\overline{x} - h,0)|) \right] - \frac{\alpha C^{\alpha , \varepsilon }_h |h|^2}{A} < 0, \end{aligned}$$

which is impossible.

**Case 2d: Proof of** (3.14). From the previous steps it follows that at least one among inequalities (3.17), (3.24), and (3.27) has to be satisfied. Recalling the definition of $f_{\varepsilon } (\alpha )$, this concludes the proof of (3.14) and, in turn, of the lemma. $\quad \square$

## Phases Separation and Optimal Regularity

As already mentioned in the Introduction, the main problem in establishing optimal regularity is that one cannot exclude *a priori* the existence of free boundary points where the function *u* changes sign. Indeed, at such points $\partial _y u (\cdot , 0)$ would be discontinuous, with a jump of $2 g' (0^+)$. This is ruled out by the next proposition, which shows that the two “phases” $\{ x \in {\mathbb {R}}^n : u (x, 0) > 0 \}$ and $\{ x \in {\mathbb {R}}^n : u (x, 0) < 0 \}$ are well separated.

### Proposition 4.1

Let $u_{\scriptscriptstyle A}$ and *g* be as in Theorem 1.1, let $u \in H^1 (\mathbb {R}^n \times (0,A))$ be a solution of (1.2), and let $x \in \partial K_u$, where $K_u$ is defined by (1.3). Then, there exists $r_0 =r_0 (x) \in (0, 1)$ such that

$$\begin{aligned} B^n_{r_0} (x) \cap \overline{\{ x' \in {\mathbb {R}}^n : u (x', 0) > 0 \}} \cap \overline{\{ x' \in {\mathbb {R}}^n : u (x', 0) < 0 \}} = \varnothing . \end{aligned}$$

Before proving Proposition 4.1, we show how this allows us to prove Theorem 1.1.

### Proof of Theorem 1.1

Let $x \in \partial K_u$. Without any loss of generality, thanks to Proposition 4.1, we can assume that

$$\begin{aligned} u (x', 0) \ge 0 \qquad \text { for every } x' \in B^n_{r_0} (x), \end{aligned}$$

where $r_0$ is given by Proposition 4.1. We claim that there exists $0 < {\widehat{r}} \le r_0$ and $D' > 0$, such that

4.1 $$\begin{aligned} D^2_{xx} u (x', y) \ge -D' \qquad \text { for every } (x', y) \in B_{{\widehat{r}}} (x, 0) \cap \{ y > 0 \}. \end{aligned}$$

Indeed, let us write $u = u_1 + u_2 + u_3$, where $u_1$, $u_2$, and $u_3$ are the harmonic functions in $\mathbb {R}^{n} \times (0,A)$ with the following boundary conditions:

$$\begin{aligned} {\left\{ \begin{array}{ll} u_1 = 0 &{}\quad \text { on } \{ y = 0 \}, \\ u_1 = u_{\scriptscriptstyle A} &{}\quad \text { on } \{ y = A \}, \end{array}\right. } \qquad {\left\{ \begin{array}{ll} u_2 = u^+ &{}\quad \text { on } \{ y = 0 \}, \\ u_2 = 0 &{}\quad \text { on } \{ y = A \}, \end{array}\right. } \qquad {\left\{ \begin{array}{ll} u_3 = u^- &{}\quad \text { on } \{ y = 0 \}, \\ u_3 = 0 &{}\quad \text { on } \{ y = A \}. \end{array}\right. } \end{aligned}$$

Note now that $u_3$ is $C^{\infty }$ in a neighborhood of (*x*, 0), since $u^- = 0$ in $B^n_{r_0} (x)$. Analogously, $u_1$ is also $C^{\infty }$ in a neighborhood of (*x*, 0). On the other hand, by maximum principle $u_2 \ge 0$. Therefore, an argument similar to the one used in the proof of Lemma 3.5 shows that, for every $y \in [0,A]$, $u_2 (\cdot , y)$ is semiconvex. Therefore,

$$\begin{aligned} D^2_{xx} u_2 (x', y) \ge - \overline{D} \quad \text { for every } (x', y) \in \mathbb {R}^n \times (0, A). \end{aligned}$$

Then, using the fact that $u_1$ and $u_3$ are smooth, (4.1) follows.

We now note that *v* defined in (1.5) is a harmonic function in $\mathbb {R}^n \times (0,A)$ satisfying

$$\begin{aligned} v\ge 0\quad \text {and}\quad v [ \partial _y v + g' (0^+) - g' (2 |v|)] \equiv 0 \qquad \text {on }\{y=0\} \cap B^n_{{\widehat{r}}} (x), \end{aligned}$$

which is just a minor variation of the classical Signorini problem $v\partial _yv=0$ [5, 6]. Thus, the remaining part of the proof of Theorem 1.1 can easily be obtained by repeating (with the needed minor modifications) the arguments used in [5, 8]. $\quad \square$

We now give the proof of Proposition 4.1.

### Proof of Proposition 4.1

Without any loss of generality, we can assume $x = 0$. We will argue by contradiction, assuming that

4.2 $$\begin{aligned} B^n_{r} \cap \overline{\{ x' \in {\mathbb {R}}^n : u (x', 0)> 0 \}} \cap \overline{\{ x' \in {\mathbb {R}}^n : u (x', 0) < 0 \}} \ne \varnothing \quad \text { for every } r > 0.\nonumber \\ \end{aligned}$$

We divide the proof into two steps.

**Step 1:** We show that (4.2) implies that $u (\cdot , 0)$ is differentiable at $x=0$ with $\nabla _x u (0,0) = 0$. Since $u (0, 0) = 0$, $u^+\ge 0$, and $u^-\le 0$, we have

$$\begin{aligned} 0 \in \partial ^-_x u^+ (0, 0) \quad \text { and } \quad 0 \in \partial ^+_x u^- (0,0), \end{aligned}$$

where we denote by $\partial ^-_x u^+ (\cdot , 0)$ and $\partial ^+_x u^- (\cdot ,0)$ the subdifferential of $u^+ (\cdot , 0)$ and the superdifferential of $u^- (\cdot , 0)$, respectively. Suppose now that (4.2) is satisfied but, by contradiction, there exists $\xi \in \mathbb {R}^n$ such that

$$\begin{aligned} \xi \in \big ( \partial ^-_x u^+ (0, 0) \cup \partial ^+_x u^- (0,0) \big ) {\setminus } \{ 0 \}. \end{aligned}$$

Without loss of generality, we can assume $\xi \in \partial ^-_x u^+ (0, 0)$ and $\xi = b {\mathbf {e}}_1$ for some $b > 0$, that is

4.3 $$\begin{aligned} b {\mathbf {e}}_1 \in \partial ^-_x u^+ (0, 0), \quad \text { for some } b > 0. \end{aligned}$$

Since, by Lemma 3.5, $u^+ (\cdot , 0)$ is semiconvex with semiconvexity constant $\overline{D}$, (4.3) implies that

4.4 $$\begin{aligned} u^+ (x, 0) + \overline{D} |x|^2 \ge u^+ (0, 0) + b {\mathbf {e}}_1 \cdot x \quad \text { for every } x\in \mathbb {R}^n. \end{aligned}$$

Setting $x_b : = \frac{b}{2 \overline{D}} {\mathbf {e}}_1$, the above inequality can be written as $u^+ (x, 0) \ge \overline{D} ( |x_b|^2 - | x - x_b|^2 )$, so that

4.5 $$\begin{aligned} u (x, 0) > 0 \quad \text { for every } x \in B^n_{|x_b|} (x_b). \end{aligned}$$

We now divide the proof of Step 1 into two substeps.

**Step 1a:** We show that

$$\begin{aligned} (4.3) \quad \Longrightarrow \quad \partial ^+_x u^- (0, 0) {\setminus } \{ 0 \} \ne \varnothing . \end{aligned}$$

Suppose, by contradiction, that (4.3) is satisfied but $\partial ^+_x u^- (0, 0) = \{ 0 \}$. Then, since $u^-$ is semiconcave, $u^- (\cdot , 0)$ is differentiable in 0 and

4.6 $$\begin{aligned} u (x, 0) \ge u^- (x, 0) \ge o (|x|) \quad \text { for every } x \in \mathbb {R}^n. \end{aligned}$$

By (4.2), we can find a sequence $\{ x_k \}_{k \in {\mathbb {N}}} \subset \mathbb {R}^n {\setminus } \{ 0 \}$ with $x_k \rightarrow 0$ such that

4.7 $$\begin{aligned} u (x_k, 0) < 0 \quad \text { for every } k \in {\mathbb {N}}. \end{aligned}$$

Setting $h_k:= 2 |x_k| {\mathbf {e}}_1$, thanks to (4.6) we have

4.8 $$\begin{aligned} u (x_k - h_k, 0 ) \ge u^- (x_k - h_k, 0 ) = o (|x_k - h_k|) = o (|x_k|), \end{aligned}$$

where the last equality follows from our choice of the sequence $\{ h_k \}_{k \in {\mathbb {N}}}$. On the other hand, by (4.4) it follows that

4.9 $$\begin{aligned}&u^+ (x_k + h_k, 0) \ge b {\mathbf {e}}_1 \cdot (x_k + h_k) - \overline{D} |x_k + h_k|^2 \nonumber \\&\quad = b |x_k| \left( 2 + {\mathbf {e}}_1 \cdot \frac{x_k}{|x_k|} \right) - \overline{D} |x_k|^2 \left( 5 + 4 {\mathbf {e}}_1 \cdot \frac{x_k}{|x_k|} \right) \nonumber \\&\quad \ge b |x_k| - 9\overline{D} |x_k|^2 \ge \frac{b}{2} |x_k|, \end{aligned}$$

for *k* sufficiently large. Thanks to Remark 3.7, combining (4.7), (4.8), and (4.9), we have that, for *k* large enough,

$$\begin{aligned} 0&> 2 u (x_k, 0 ) \ge 2 u^- (x_k, 0 ) \\&\ge \left[ u (x_k + h_k, 0 ) + u (x_k - h_k, 0 ) - \overline{C} |h_k|^2 \right] ^- \\&\ge \left[ \frac{b}{2} |x_k| + o (|x_k|) - 4 \overline{C} |x_k|^2 \right] ^- = 0, \end{aligned}$$

which is impossible.

**Step 1b:** We conclude the proof of Step 1. By Step 1a, there exists $d > 0$ and ${\mathbf {e}} \in {\mathbb {S}}^{n} \cap \{ y = 0 \}$ such that $d {\mathbf {e}} \in \partial ^+_x u^- (0,0)$. Since, by Lemma 3.6, $u^- (\cdot , 0)$ is semiconcave with semiconcavity constant $\overline{C}$, by repeating the same argument used to show (4.4) we have that

4.10 $$\begin{aligned} u (x, 0) < 0 \qquad \text { for every } x \in B^n_{|x_{d}|} (x_{d}), \end{aligned}$$

where we set $x_d : = \frac{d}{2 \overline{C}} {\mathbf {e}}$. Taking into account (4.5), this implies ${\mathbf {e}} = - {\mathbf {e}}_1$, thus $x_d = - \frac{d}{2 \overline{C}} {\mathbf {e}}_1$. We will now show that $\partial _{x_1} u (\cdot , 0)$ is unbounded, against Lemma 3.4.

To this aim, for every $\varepsilon > 0$ we set $w_{\varepsilon }:= - \varepsilon {\mathbf {e}}_1$. In this way, $w_\varepsilon \rightarrow 0$ as $\varepsilon \rightarrow 0^+$ and $w_{\varepsilon } \in B^n_{|x_{d}|} (x_{d})$ for $\varepsilon$ sufficiently small, so that $u (w_\varepsilon ,0 ) < 0$. We claim that

4.11 $$\begin{aligned} \lim _{\varepsilon \rightarrow 0^+} \partial _{x_1} u (w_{\varepsilon },0) = + \infty . \end{aligned}$$

Let ${\tilde{u}}: \mathbb {R}^n \times [0, \infty ) \rightarrow \mathbb {R}$ be the harmonic extension of *u*(*x*, 0) to the half space $\mathbb {R}^n \times [0, \infty )$. We have

$$\begin{aligned}&\frac{1}{C_n} \partial _{x_1} u (w_{\varepsilon },0) = \frac{1}{C_n} \partial _{x_1} {\tilde{u}} (w_{\varepsilon },0) = \int _{{\mathbb {R}}^n} \frac{((w_{\varepsilon })_1 - z_1)}{|w_{\varepsilon } - z|^{n+1}} \left( \partial _y {\tilde{u}} (w_{\varepsilon }, 0) - \partial _y {\tilde{u}} (z, 0) \right) \, \mathrm{d}z \\&\quad = \int _{{\mathbb {R}}^n} \frac{((w_{\varepsilon })_1 - z_1)}{|w_{\varepsilon } - z|^{n+1}} \left( \partial _y ({\tilde{u}} -u) (w_{\varepsilon }, 0) - \partial _y ({\tilde{u}} -u)(z, 0) \right) \, \mathrm{d}z \\&\qquad +\, \int _{{\mathbb {R}}^n} \frac{((w_{\varepsilon })_1 - z_1)}{|w_{\varepsilon } - z|^{n+1}} \left( \partial _y u (w_{\varepsilon }, 0) - \partial _y u (z, 0) \right) \, \mathrm{d}z, \end{aligned}$$

for some positive dimensional constant $C_n$. Since ${\tilde{u}} - u$ vanishes on $\{ y = 0 \}$ and is harmonic in $\mathbb {R}^n \times (0,A)$, we have $x \mapsto \partial _y ({\tilde{u}} -u) (x, 0) \in C^{\infty } (\mathbb {R}^n)$. Therefore, to prove our claim it will be sufficient to show that the last integral diverges as $\varepsilon \rightarrow 0^+$. Let $f : \mathbb {R}^n \rightarrow {\mathbb {R}}$ be defined as

$$\begin{aligned} f (x) : = {\left\{ \begin{array}{ll} \partial _y u (x, 0) &{}\quad \text { if } u (x, 0) < 0, \\ - g' (0^+) &{}\quad \text { if } u (x, 0) \ge 0. \end{array}\right. } \end{aligned}$$

Since $\partial _y u (x, 0)=-g'(2|u (x, 0)|)$ where $u (x, 0) < 0$, it follows that that *f* is Lipschitz continuous, with Lipschitz constant $2 \Vert g '' \Vert _{L^{\infty }} \frac{L_A}{1-2A\Vert g '' \Vert _{L^{\infty }}}$ (recall Lemma 3.4). In what follows, we set

4.12 $$\begin{aligned} \lambda := \min \left\{ \frac{b}{\overline{D}} , \frac{d}{\overline{C}} \right\} . \end{aligned}$$

Given $r > 0$ with $0< r < \lambda / 4$, we split the integral under consideration as follows:

$$\begin{aligned}&\int _{{\mathbb {R}}^n} \frac{((w_{\varepsilon })_1 - z_1)}{|w_{\varepsilon } - z|^{n+1}} \left( \partial _y u (w_{\varepsilon }, 0) - \partial _y u (z, 0) \right) \, \mathrm{d}z \\&\quad = \int _{{\mathbb {R}}^n {\setminus } B^n_r} \frac{((w_{\varepsilon })_1 - z_1)}{|w_{\varepsilon } - z|^{n+1}} \left( \partial _y u (w_{\varepsilon }, 0) - \partial _y u (z, 0) \right) \, \mathrm{d}z. \\&\qquad + \int _{B^n_r} \frac{((w_{\varepsilon })_1 - z_1)}{|w_{\varepsilon } - z|^{n+1}} \left( \partial _y u (w_{\varepsilon }, 0) - \partial _y u (z, 0) \right) \, \mathrm{d}z. \end{aligned}$$

We can disregard the first integral, which is bounded for $\varepsilon$ small enough. Concerning the second integral, using the fact that $u (w_{\varepsilon }, 0) < 0$, we have

$$\begin{aligned}&\int _{B^n_r} \frac{((w_{\varepsilon })_1 - z_1)}{|w_{\varepsilon } - z|^{n+1}} \left( \partial _y u (w_{\varepsilon }, 0) - \partial _y u (z, 0) \right) \, \mathrm{d}z = \int _{B^n_r} \frac{((w_{\varepsilon })_1 - z_1)}{|w_{\varepsilon } - z|^{n+1}} \left( \partial _y u (w_{\varepsilon }, 0) - f (w_{\varepsilon }) \right) \, \mathrm{d}z \\&+ \int _{B^n_r} \frac{((w_{\varepsilon })_1 - z_1)}{|w_{\varepsilon } - z|^{n+1}} \left( f (z) - \partial _y u (z, 0) \right) \, \mathrm{d}z + \int _{B^n_r} \frac{((w_{\varepsilon })_1 - z_1)}{|w_{\varepsilon } - z|^{n+1}} \left( f (w_{\varepsilon }) - f (z) \right) \, \mathrm{d}z \\&= \int _{B^n_r} \frac{((w_{\varepsilon })_1 - z_1)}{|w_{\varepsilon } - z|^{n+1}} \left( f (z) - \partial _y u (z, 0) \right) \, \mathrm{d}z + \int _{B^n_r} \frac{((w_{\varepsilon })_1 - z_1)}{|w_{\varepsilon } - z|^{n+1}} \left( f (w_{\varepsilon }) - f (z) \right) \, \mathrm{d}z \\&=: I_1^{\varepsilon } + I_2^{\varepsilon }. \end{aligned}$$

Since *f* is Lipschitz continuous, $I_2^{\varepsilon }$ is uniformly bounded in $\varepsilon$. By definition of *f* and by (4.5), we can split $I_1^{\varepsilon }$ in the following way:

$$\begin{aligned}&I_1^{\varepsilon } =\int _{B^n_r} \frac{((w_{\varepsilon })_1 - z_1)}{|w_{\varepsilon } - z|^{n+1}} \left( f (z) - \partial _y u (z, 0) \right) \, \mathrm{d}z \\&\quad = \int _{B^n_r \cap \{ u \ge 0 \}} \frac{((w_{\varepsilon })_1 - z_1)}{|w_{\varepsilon } - z|^{n+1}} \left( f (z) - \partial _y u (z, 0) \right) \, \mathrm{d}z \\&\quad = \int _{B^n_r \cap B^n_{|x_b|} (x_b)} \frac{((w_{\varepsilon })_1 - z_1)}{|w_{\varepsilon } - z|^{n+1}} \left( f (z) - \partial _y u (z, 0) \right) \, \mathrm{d}z \\&\qquad + \int _{B^n_r \cap \{ u \ge 0 \} {\setminus } B^n_{|x_b|} (x_b)} \frac{((w_{\varepsilon })_1 - z_1)}{|w_{\varepsilon } - z|^{n+1}} \left( f (z) - \partial _y u (z, 0) \right) \, \mathrm{d}z = : I_{1,1}^{\varepsilon } + I_{1,2}^{\varepsilon }. \end{aligned}$$

We claim that $I_{1,2}^{\varepsilon }$ is uniformly bounded in $\varepsilon$. Indeed, first of all we observe that, thanks to (2.1) and Lemma 3.4,

$$\begin{aligned}&| f (z) - \partial _y u (z, 0) | = g' (0^+) + \partial _y u (z, 0) \le g' (0^+) + g' (2 u (z, 0)) \\&\le 2 \big ( \, g' (0^+) + \Vert g '' \Vert _{L^{\infty }} u (z, 0) \, \big ) \le 2 \left( g' (0^+) + r \frac{L_A \Vert g '' \Vert _{L^{\infty }}}{1-2A\Vert g '' \Vert _{L^{\infty }}} \right) =: c_r \end{aligned}$$

for every $z \in B^n_r \cap \{ u \ge 0 \}$. Then, recalling (4.10), we have

$$\begin{aligned} | I_{1,2}^{\varepsilon } |&\le c_r \int _{B^n_r \cap \{ u \ge 0 \} {\setminus } B^n_{|x_b|} (x_b)} \frac{1}{|w_{\varepsilon } - z|^{n}} \, \mathrm{d}z = c_r \int _{B^n_r {\setminus } ( B^n_{|x_b|} (x_b) \cup B^n_{|x_d|} (x_d)) } \frac{1}{|w_{\varepsilon } - z|^{n}} \, \mathrm{d}z \\&= c_r \int _0^r \int _{{\mathbb {S}}^{n-1} \cap \{ \omega \in {\mathbb {S}}^{n-1} :- \rho \, \overline{C} / d< \omega _1 < \rho \overline{D} / b \}} \frac{\rho ^{n-1}}{|w_{\varepsilon } - \rho \omega |^{n}} \,\mathrm{d}{\mathcal {H}}^{n-1} (\omega ) \, d \rho \\&\le c_r \int _0^r \int _{\Sigma _{\rho }} \frac{\rho ^{n-1}}{|w_{\varepsilon } - \rho \omega |^{n}} \, \mathrm{d}{\mathcal {H}}^{n-1} (\omega ) \, d \rho , \end{aligned}$$

where we set

$$\begin{aligned} \Sigma _{\rho } : = \left\{ \omega \in {\mathbb {S}}^{n-1} : |\omega _1| < \frac{\rho }{\lambda } \right\} , \end{aligned}$$

and $\lambda$ is defined by (4.12). Since $w_{\varepsilon }:= - \varepsilon {\mathbf {e}}_1$, we note that for every $\rho \in (0,r)$ and $\omega \in \Sigma _{\rho }$

$$\begin{aligned} |w_{\varepsilon } - \rho \, \omega |^2 = \rho ^2 + 2 \varepsilon \rho \, \omega _1 + \varepsilon ^2> \rho ^2 - 2 \varepsilon \rho ^2 \, \lambda + \varepsilon ^2 = \rho ^2 \left( 1 - 2 \varepsilon \lambda \right) + \varepsilon ^2 > \frac{1}{2} \rho ^2, \end{aligned}$$

for $\varepsilon$ sufficiently small. Therefore, for $\varepsilon$ sufficiently small we obtain

$$\begin{aligned} | I_{1,2}^{\varepsilon } |&\le 2^{\frac{n}{2}} c_r \int _0^r \int _{\Sigma _{\rho }} \frac{1}{\rho } \, \mathrm{d}{\mathcal {H}}^{n-1} (\omega ) \, d \rho \le 2^{\frac{n}{2}} c_r \, C, \end{aligned}$$

where we used the fact that ${\mathcal {H}}^{n-1} (\Sigma _{\rho }) \le C \rho$ for some positive constant $C = C (n)$.

Let us now estimate $I_1^{\varepsilon }$. Since $z_1 > 0$ for every $z \in B^n_r \cap B^n_{|x_b|} (x_b)$ and $(w_{\varepsilon })_1 = - \varepsilon < 0$, we have $(w_{\varepsilon })_1 - z_1 < 0$. Therefore, since $g'>0$,

$$\begin{aligned} I_{1,1}^{\varepsilon }&= \int _{B^n_r \cap B^n_{|x_b|} (x_b)} \frac{(w_{\varepsilon })_1 - z_1}{|w_{\varepsilon } - z|^{n+1}} \left( f (z) - \partial _y u (z, 0) \right) \, \mathrm{d}z \\&= \int _{B^n_r \cap B^n_{|x_b|} (x_b)} \frac{z_1 - (w_{\varepsilon })_1 }{|w_{\varepsilon } - z|^{n+1}} \left( g' (0^+) + g' (2 u (z, 0) \right) \, \mathrm{d}z \\&\ge g' (0^+) \int _{B^n_r \cap B^n_{|x_b|} (x_b)} \frac{z_1 - (w_{\varepsilon })_1 }{|w_{\varepsilon } - z|^{n+1}} \, \mathrm{d}z \ge g' (0^+) \int _{B^n_{r- \varepsilon } (w_{\varepsilon }) \cap B^n_{|x_b|} (x_b)} \frac{z_1 - (w_{\varepsilon })_1 }{|w_{\varepsilon } - z|^{n+1}} \, \mathrm{d}z \\&= g' (0^+) \int _{B^n_{r- \varepsilon } \cap B^n_{|x_b|} (x_b - w_{\varepsilon })} \frac{\tau _1}{|\tau |^{n+1}} \, d \tau = g' (0^+) \int _{\varepsilon }^{r - \varepsilon } \frac{1}{\rho } \int _{\Sigma ^{\varepsilon }_{\rho }} \omega _1 \, \mathrm{d}{\mathcal {H}}^{n-1} (\omega ) \, d \rho , \end{aligned}$$

where

$$\begin{aligned} \Sigma ^{\varepsilon }_{\rho } := \left\{ \omega \in {\mathbb {S}}^{n-1}: \frac{\overline{D} (\rho ^2 + \varepsilon ^2) + \varepsilon b}{\rho (b + 2 \varepsilon \overline{D})}< \omega _1 < 1 \right\} . \end{aligned}$$

Note now that, for $\varepsilon$ sufficiently small, since $\rho< r - \varepsilon < \lambda / 4 \le b/(4\overline{D})$, we have

$$\begin{aligned} \frac{\overline{D} (\rho ^2 + \varepsilon ^2) + \varepsilon b}{\rho (b + 2 \varepsilon \overline{D})}< 2 \frac{\overline{D} \rho }{b} < \frac{1}{2}. \end{aligned}$$

Therefore,

$$\begin{aligned} I_{1,1}^{\varepsilon }&\ge g' (0^+) \int _{\varepsilon }^{r - \varepsilon } \frac{1}{\rho } \int _{{\mathbb {S}}^{n-1} \cap \{ 1/2< \omega _1 < 1 \}} \omega _1 \, \mathrm{d}{\mathcal {H}}^{n-1} (\omega ) \, d \rho \\&= c_n \, g' (0^+) \int _{\varepsilon }^{r - \varepsilon } \frac{1}{\rho } \, d \rho = c_n \, g' (0^+) \ln \left( \frac{r - \varepsilon }{\varepsilon } \right) , \end{aligned}$$

for some positive dimensional constant $c_n$. Taking the limit as $\varepsilon \rightarrow 0^+$ we obtain

$$\begin{aligned} \lim _{\varepsilon \rightarrow 0^+} I_{1,1}^{\varepsilon } = + \infty , \end{aligned}$$

which proves (4.11). As noted before, this contradicts Lemma 3.4, concluding the proof of Step 1.

**Step 2:** We show that there exist positive constants $\gamma$, $\eta ,$ and $\overline{r}$ such that

4.13 $$\begin{aligned} \partial _y u \ge \frac{3}{4} g' (0^+) \qquad \text { in } R_{\gamma \overline{r}} := B_{\gamma \overline{r}}^n \times \left[ (1 - \gamma ) \eta \overline{r} , (1 + \gamma ) \eta \overline{r} \right] . \end{aligned}$$

By Step 1 we know that $u (\cdot , 0)$ is differentiable at $x=0$ with $\nabla _x u (0,0) = 0$, hence there exists a continuous function $\sigma : [0, \infty ) \rightarrow [0, \infty )$ with $\sigma (0) = 0$ such that

$$\begin{aligned} | u (x , 0) | \le \sigma (|x|) |x| \qquad \text { for every } x \in \mathbb {R}^n. \end{aligned}$$

Note that, with no loss of generality, we can assume that $\sigma (r)\ge r$ for all *r*.

Let $M, C, \eta$ be positive constants to be chosen later, and for every $r \in (0,1)$ set

$$\begin{aligned} \Gamma _r := B_r^n \times \left[ 0, \eta r \right] . \end{aligned}$$

We consider the harmonic function $V^+: \Gamma _r \rightarrow {\mathbb {R}}$ defined as

$$\begin{aligned} V^+ (x, y):= u (x, y) - M \frac{\sigma (r)}{r} |x - x^+_0|^2 + n M \frac{\sigma (r)}{r} y^2 - (g' (0^+) - C r) y, \end{aligned}$$

where $x^+_0 \in {\mathbb {R}}^n$ is such that $u (x^+_0 , 0) > 0$ and $|x^+_0| = c_0 r$ with $0 < c_0 \ll 1$ (note that that such a point $x^+_0$ exists, because we are assuming, by contradiction, that (0, 0) is a boundary point both for $\{ u > 0\}$ and for $\{ u < 0\}$). Since $V^+$ is harmonic, we have

$$\begin{aligned} 0 < \max _{{\overline{\Gamma }}_r} V^+ = \max _{\partial \Gamma _r } V^+, \end{aligned}$$

where the positivity comes from the fact that $V^+ (x^+_0,0) = u (x^+_0,0) > 0$. We now have several possibilities.

**Case 2a:** We show that, if *C* is sufficiently large, then there exists no $\overline{x} \in \partial \Gamma _r \cap \{ y = 0\}$ such that

$$\begin{aligned} \max _{{\overline{\Gamma }}_r} V^+ = V^+ (\overline{x}, 0). \end{aligned}$$

Suppose that such $\overline{x}$ exists. Then, it cannot be $u (\overline{x}, 0) \le 0$, since in that case

$$\begin{aligned} V^+ (\overline{x}, 0) = u (\overline{x}, 0) - M \frac{\sigma (r)}{r} | \overline{x} - x^+_0|^2 \le 0. \end{aligned}$$

Therefore, $$u (\overline{x}, 0) > 0$$, and

$$\begin{aligned} \partial _y V^+ (\overline{x}, 0)&= \partial _y u (\overline{x}, 0) - g' (0^+) + C r = g' (2 u (\overline{x}, 0)) - g' (0^+) + C r \\&\ge - 2 u (\overline{x}, 0) \Vert g'' \Vert _{L^{\infty }} + C r \ge - 2 \sigma (r) \, r \Vert g'' \Vert _{L^{\infty }} + C r. \end{aligned}$$

If we choose *C* large enough we obtain $\partial _y V^+ (\overline{x}, 0) > 0$, which is impossible.

**Case 2b:** We show that, if *M* is sufficiently large and $\eta$ is sufficiently small, then there exists no point $(\overline{x}, \overline{y})$ with $|\overline{x}| = r$ and $\overline{y} \in [0, \eta r]$ such that

$$\begin{aligned} \max _{{\overline{\Gamma }}_r} V^+ = V^+ (\overline{x}, \overline{y}). \end{aligned}$$

Indeed, suppose that such a point $(\overline{x}, \overline{y})$ exists. Then, since $|\overline{x}| = r$ and $|x_0^+ | = c_0 r$, we have

$$\begin{aligned} (1 - c_0)^2 r^2 \le |\overline{x} - x^+_0|^2 \le (1 + c_0)^2 r^2. \end{aligned}$$

Thus,

$$\begin{aligned} 0&< V^+ (\overline{x}, \overline{y}) = u (\overline{x}, \overline{y}) - M \frac{\sigma (r)}{r} |\overline{x} - x^+_0|^2 + n M \frac{\sigma (r)}{r} \overline{y}^2 - (g' (0^+) - C r) \overline{y} \\&\le u (\overline{x}, \overline{y}) - M \, (1 - c_0)^2 \sigma (r) r + n M \frac{\sigma (r)}{r} \overline{y}^2 - (g' (0^+) - C r) \overline{y}, \end{aligned}$$

so that (recall that $c_0\ll 1$)

$$\begin{aligned} u (\overline{x}, \overline{y})&> M \, (1 - c_0)^2 \sigma (r) r - n M \frac{\sigma (r)}{r} \overline{y}^2 + (g' (0^+) - C r) \overline{y} \\&\ge M \, (1 - c_0)^2 \sigma (r) \, r - ( n M \, \eta ^2 \, \sigma (r) \, r + C \eta r^2 ) + g' (0^+) \overline{y} \\&\ge \frac{M}{2} \sigma (r) \, r + g' (0^+) \overline{y}, \end{aligned}$$

for $\eta$ small enough. Thanks to (3.10), this last estimate gives

$$\begin{aligned}&\frac{M}{2} \sigma (r) \, r + g' (0^+) \overline{y} \\&\quad \le u (\overline{x}, \overline{y}) \le u (\overline{x}, 0) + g' (0^+) \, \overline{y} + C_1 \, \frac{\overline{y}^2}{2} \le \sigma (r) \, r + C_1 \, \eta ^2 \frac{r^2}{2} + g' (0^+) \, \overline{y}, \end{aligned}$$

which is impossible for *M* sufficiently large.

**Case 2c:** We show that, if *M* is sufficiently large and $\eta$ is sufficiently small, then there exists no point $\overline{x} \in {\mathbb {R}}^n$ with $r/2 \le |\overline{x}| \le r$ such that

$$\begin{aligned} \max _{{\overline{\Gamma }}_r} V^+ = V^+ (\overline{x}, \eta r). \end{aligned}$$

This case can be treated as Case 2b.

**Case 2d:** We show that, if *M* is sufficiently large and $\eta$ is sufficiently small, there exist $\gamma , \overline{r} > 0$ such that (4.13) is satisfied. From Cases 2a–2c, there exists $\overline{x} \in {\mathbb {R}}^n$ with $|\overline{x}| < r/2$ such that

$$\begin{aligned} \max _{{\overline{\Gamma }}_r} V^+ = V^+ (\overline{x}, \eta r), \end{aligned}$$

so that

$$\begin{aligned} 0&\le \partial _y V^+ (\overline{x}, \eta r) = \partial _y u (\overline{x}, \eta r) +2 n M \eta \, \sigma (r) - (g' (0^+) - C r). \end{aligned}$$

Using the fact that $r \le \sigma (r)$ we have

4.14 $$\begin{aligned} \partial _y u (\overline{x}, \eta r) \ge g' (0^+) - 2 n M \eta \, \sigma (r) - C r \ge g' (0^+) - C_{\eta } \, \sigma ( r ), \end{aligned}$$

where $C_{\eta } = 2 n M \eta + C$. For $\gamma \in (1/2,1)$ let us set

$$\begin{aligned} (\overline{x}, \eta r) \in R_{\gamma r}:= B_{\gamma r}^n \times \left[ (1 - \gamma ) \eta r , (1 + \gamma ) \eta r \right] . \end{aligned}$$

Thanks to (3.10), the function $g' (0^+) + 2 C_1 \eta r - \partial _y u$ is harmonic and nonnegative in $\Gamma _{2 r}$. Thus, by (4.14) and Harnack inequality, there exists a constant $C_{\gamma } > 0$ such that

$$\begin{aligned} \sup _{R_{\gamma r} } \left( g' (0^+) + 2 C_1 \eta r - \partial _y u \right)\le & {} C_{\gamma } \inf _{R_{\gamma r}} \left( g' (0^+) + 2 C_1 \eta r - \partial _y u \right) \\\le & {} C_{\gamma } \left( g' (0^+) + 2 C_1 \eta r - (g' (0^+) - C_{\eta } \, \sigma ( r )) \right) \\= & {} C_{\gamma } ( 2 C_1 \eta r + C_{\eta } \, \sigma ( r ) ). \end{aligned}$$

From the previous chain of inequalities we obtain

$$\begin{aligned} \partial _y u \ge g' (0^+) + 2 C_1 (1 - C_{\gamma }) \eta r - C_{\gamma } C_{\eta } \, \sigma ( r ) \qquad \text { in } R_{\gamma r}. \end{aligned}$$

Therefore, there exists $\overline{r} = \overline{r} (\gamma )$ such that

$$\begin{aligned} \partial _y u \ge \frac{3}{4} g' (0^+) \qquad \text { in } R_{\gamma \overline{r}}, \end{aligned}$$

thus showing (4.13).

**Step 3:** We conclude. An argument analogous to that one used in Step 2 can be applied to the harmonic function $V^-: \Gamma _r \rightarrow {\mathbb {R}}$ defined as

$$\begin{aligned} V^- (x, y):= u (x, y) + M \frac{\sigma (r)}{r} |x - x^-_0|^2 - n M \frac{\sigma (r)}{r} y^2 + (g' (0^+) - C r) y, \end{aligned}$$

where $x^-_0 \in {\mathbb {R}}^n$ is such that $u (x^-_0 , 0) < 0$ and $|x^-_0| = c^-_0 r$ with $0 < c^-_0 \ll 1$, to obtain that

$$\begin{aligned} \partial _y u \le - \frac{3}{4} g' (0^+) \qquad \text { in } R_{\gamma \overline{r}}. \end{aligned}$$

Comparing the inequality above to (4.13), we obtain the desired contradiction. $\quad \square$

## Frequency Formula

In this section we prove a frequency formula, which will allow us to study the blow up profiles of solutions *u* of (1.2). To this purpose, assuming that (0, 0) is a free boundary point for *u*, and that $u (x, 0) \ge 0$ in a neighborhood of 0 (cf. Proposition 4.1), we investigate the regularity properties of the function $v: {\mathbb {R}}^n \times [-A,A] \rightarrow {\mathbb {R}}$ defined by (1.5).

Throughout this section we assume that the hypotheses of Theorem 1.1 are satisfied, that *v* is given by (1.5) where *u* is a solution of (1.2), that (0, 0) is a free boundary point for *v*, and that $v (x, 0) \ge 0$ for every $x \in B^n_{r_0}$, where $r_0$ is given by Proposition 4.1. Therefore, *v* satisfies:

$$\begin{aligned} {\left\{ \begin{array}{ll} \Delta v = 0 \, &{}\quad \text { in } \, B_{r_0} {\setminus } \{ y = 0 \}, \\ v \ge 0 \, &{}\quad \text { on }\, B_{r_0}^n, \\ \partial _{y} v \le g' (2 v) - g' (0^+) \, &{}\quad \text { on }\, B_{r_0}^n, \\ \partial _{y} v = g' (2 v) - g' (0^+) \, &{}\quad \text { on }\, B_{r_0}^n \cap \{ v > 0 \}. \end{array}\right. } \end{aligned}$$

Since *v* is even with respect to the hyperplane $\{ y = 0 \}$, we have

5.1 $$\begin{aligned} v_{\scriptscriptstyle RT} = v_{\scriptscriptstyle LT} \quad \text { and } \quad - \frac{\partial v_{\scriptscriptstyle LT}}{\partial y} = \frac{\partial v_{\scriptscriptstyle RT}}{\partial y} \qquad \text { on } B^n_{r_0}. \end{aligned}$$

First of all we observe that

$$\begin{aligned} \partial _{y} v (x, 0 )= g' (2 v (x, 0 )) - g' (0^+) \le 2 \Vert g '' \Vert v (x, 0 ) \le \frac{2 \Vert g '' \Vert L_A}{1-2A\Vert g '' \Vert _{L^{\infty }}} r_0 = : C_0, \end{aligned}$$

for every $x \in B^n_{r_0}$, where we used the definition of *v*, Lemma 3.4, and the fact that (0, 0) is a free boundary point. From the above inequality it follows that the function

5.2 $$\begin{aligned} {\widetilde{v}} (x, y):= v (x, y) - C_0 |y| \end{aligned}$$

is superharmonic in $B_{r_0}$. Indeed, let $\varphi \in C^{\infty }_c (B_{r_0})$ with $\varphi \ge 0$. Then, using the fact that ${\widetilde{v}}$ is harmonic in $B_{r_0} {\setminus } \{ y = 0\}$

$$\begin{aligned}&\int _{B_{r_0}} {\widetilde{v}} \Delta \varphi \, \mathrm{d}z = \int _{B_{r_0} \cap \{ y> 0\}} {\widetilde{v}} \Delta \varphi \, \mathrm{d}z + \int _{B_{r_0} \cap \{ y< 0\}} {\widetilde{v}} \Delta \varphi \, \mathrm{d}z\\&\quad = \int _{B_{r_0} \cap \{ y> 0\}} \text {div} ( {\widetilde{v}} \, \nabla \varphi ) \, \mathrm{d}z + \int _{B_{r_0} \cap \{ y> 0\}} \text {div} ( {\widetilde{v}} \, \nabla \varphi ) \, \mathrm{d}z \\&\qquad - \int _{B_{r_0} \cap \{ y> 0\}} \nabla {\widetilde{v}} \cdot \nabla \varphi \, \mathrm{d}z - \int _{B_{r_0} \cap \{ y< 0\}} \nabla {\widetilde{v}} \cdot \nabla \varphi \, \mathrm{d}z\\&\quad = \int _{B_{r_0}^n} ( {\widetilde{v}}_{\scriptscriptstyle LT} - {\widetilde{v}}_{\scriptscriptstyle RT} ) \frac{\partial \varphi }{\partial y} \, \mathrm{d}{\mathcal {H}}^n - \int _{B_{r_0} \cap \{ y > 0\}} \text {div} ( \varphi \nabla {\widetilde{v}} ) \, \mathrm{d}z - \int _{B_{r_0} \cap \{ y < 0\}} \text {div} ( \varphi \nabla {\widetilde{v}} ) \, \mathrm{d}z \\&\quad = \int _{B^n_{r_0}} \varphi \left( \frac{\partial {\widetilde{v}}_{\scriptscriptstyle RT}}{\partial y} - \frac{\partial {\widetilde{v}}_{\scriptscriptstyle LT}}{\partial y} \right) = 2 \int _{B^n_{r_0}} \varphi \frac{\partial {\widetilde{v}}_{\scriptscriptstyle RT}}{\partial y} \, \mathrm{d}{\mathcal {H}}^n \le 0, \end{aligned}$$

where we used (5.1). We can now state the main result of the section.

### Proposition 5.1

Let $F_v : (0, \infty ) \rightarrow [0, \infty )$ be given by

$$\begin{aligned} F_v (r):= \int _{\partial B_r} v^2 \, \mathrm{d}{\mathcal {H}}^n, \end{aligned}$$

let $r_0$ be given by Proposition 4.1, and set

$$\begin{aligned} \Phi _v (r):= r \frac{d}{dr} \log \left( \max \{ F_v (r) , r^{n+4} \} \right) . \end{aligned}$$

Then, there exist $0 < \overline{r}_0 \le r_0$, and a positive constant *C*, such that the function $r \mapsto \Phi _v (r) e^{C r}$ is monotone nondecreasing for $r \in (0,\overline{r}_0)$. In particular, there exists

$$\begin{aligned} \Phi _v (0^+) = \lim _{r \rightarrow 0^+} \Phi _v (r). \end{aligned}$$

Before giving the proof, we need several auxiliary lemmas. When integrating along the boundary of a smooth $(n+1)$-dimensional set, we will denote by $\nu$ the outer unit normal, and by $v_{\nu }$ the derivative of *v* along $\nu$. We will denote the tangential gradient of *v* by $\nabla _{\tau } v$, so that $\nabla _{\tau } v = \nabla v - v_{\nu } \nu$.

The next lemma is an adaptation of [9, Lemma 7.8].

### Lemma 5.2

For every $$r \in (0, r_0)$$

$$\begin{aligned} (n-1) \int _{B_r} |\nabla v|^2 \, \mathrm{d}z&= r \int _{\partial B_r} \left[ |\nabla _{\tau } v |^2 - v_{\nu }^2 \right] \, \mathrm{d}{\mathcal {H}}^n\\&+ 4 \int _{B^n_r} ( g' (2 v) - g' (0^+) )(x \cdot \nabla _{\tau } v) \, \mathrm{d}x. \end{aligned}$$

### Proof

Since $\Delta v=0$ in $B_{r_0} {\setminus } \{ y = 0 \}$, there we have

$$\begin{aligned}&\text {div} \left[ |\nabla v|^2 z - 2 (z \cdot \nabla v) \nabla v \right] \\&= (n+1) |\nabla v|^2 + 2 (D^2 v \cdot \nabla v) \cdot z - 2 (z \cdot \nabla v) \Delta v - 2 \nabla v \cdot \left( \nabla v + (D^2 v \cdot z) \right) \\&= (n+1) |\nabla v|^2 - 2 |\nabla v|^2 = (n-1) |\nabla v|^2. \end{aligned}$$

Then,

5.3 $$\begin{aligned}&(n-1) \int _{B_r} |\nabla v|^2 \, \mathrm{d}z = \int _{B_r \cap \{ y> 0\} } \text {div} \left[ |\nabla v|^2 z - 2 (z \cdot \nabla v) \nabla v \right] \, \mathrm{d}z \nonumber \\&\qquad + \int _{B_r \cap \{ y< 0\} } \text {div} \left[ |\nabla v|^2 z - 2 (z \cdot \nabla v) \nabla v \right] \, \mathrm{d}z \nonumber \\&\quad = \int _{ \partial (B_r \cap \{ y > 0\}) } \left[ |\nabla v|^2 (z \cdot \nu ) - 2 (z \cdot \nabla v) (\nabla v \cdot \nu ) \right] \, \mathrm{d}{\mathcal {H}}^n \nonumber \\&\qquad + \int _{ \partial (B_r \cap \{ y < 0\}) } \left[ |\nabla v|^2 (z \cdot \nu ) - 2 (z \cdot \nabla v) (\nabla v \cdot \nu ) \right] \, \mathrm{d}{\mathcal {H}}^n. \end{aligned}$$

Recalling that $z = r \nu$ on $\partial B_r$, we get

5.4 $$\begin{aligned}&\int _{ \partial (B_r \cap \{ y> 0\}) } \left[ |\nabla v|^2 (z \cdot \nu ) - 2 (z \cdot \nabla v) (\nabla v \cdot \nu ) \right] \, \mathrm{d}{\mathcal {H}}^n \nonumber \\&\quad = \int _{\partial B_r \cap \{ y> 0\}} \left[ |\nabla v|^2 (z \cdot \nu ) - 2 (z \cdot \nabla v) (\nabla v \cdot \nu ) \right] \, \mathrm{d}{\mathcal {H}}^n \nonumber \\&\qquad + \int _{B^n_r} \left[ |\nabla v_{\scriptscriptstyle RT}|^2 (x \cdot \nu ) - 2 (x \cdot \nabla v_{\scriptscriptstyle RT}) (\nabla v_{\scriptscriptstyle RT} \cdot \nu ) \right] \, \mathrm{d}x \nonumber \\&\quad = r \int _{\partial B_r \cap \{ y> 0\}} \left[ |\nabla v|^2 - 2 v_{\nu }^2 \right] \, \mathrm{d}{\mathcal {H}}^n\nonumber \\&\quad + \int _{B^n_r} \left[ |\nabla v_{\scriptscriptstyle RT}|^2 (x \cdot \nu ) - 2 (x \cdot \nabla v_{\scriptscriptstyle RT}) (\nabla v_{\scriptscriptstyle RT} \cdot \nu ) \right] \, \mathrm{d}x \nonumber \\&\quad = r \int _{\partial B_r \cap \{ y> 0\}} \left[ | \nabla _{\tau } v |^2 - v_{\nu }^2 \right] \, \mathrm{d}{\mathcal {H}}^n + \int _{B^n_r} 2 (x \cdot \nabla v_{\scriptscriptstyle RT}) \, \partial _{y} v_{\scriptscriptstyle RT} \, \mathrm{d}x \nonumber \\&\quad = r \int _{\partial B_r \cap \{ y > 0\}} \left[ | \nabla _{\tau } v |^2 - v_{\nu }^2 \right] \, \mathrm{d}{\mathcal {H}}^n + \int _{B^n_r} 2 (x \cdot \nabla _{x} v_{\scriptscriptstyle RT}) \, \partial _{y} v_{\scriptscriptstyle RT} \, \mathrm{d}x. \end{aligned}$$

Similarly,

5.5 $$\begin{aligned}&\int _{ \partial (B_r \cap \{ y< 0\}) } \left[ |\nabla v|^2 (z \cdot \nu ) - 2 (z \cdot \nabla v) (\nabla v \cdot \nu ) \right] \, \mathrm{d}{\mathcal {H}}^n \nonumber \\&\quad = r \int _{\partial B_r \cap \{ y < 0\}} \left[ | \nabla _{\tau } v |^2 - v_{\nu }^2 \right] \, \mathrm{d}{\mathcal {H}}^n - \int _{B^n_r} 2 (x \cdot \nabla _{x} v_{\scriptscriptstyle LT}) \, \partial _{y} v_{\scriptscriptstyle LT} \, \mathrm{d}x. \end{aligned}$$

Combining (5.3), (5.4), and (5.5) we obtain

$$\begin{aligned}&(n-1) \int _{B_r} |\nabla v|^2 \, \mathrm{d}z = r \int _{\partial B_r} \left[ | \nabla _{\tau } v |^2 - v_{\nu }^2 \right] \, \mathrm{d}{\mathcal {H}}^n\\&\quad + 2 \int _{B^n_r} \left[ (x \cdot \nabla _{x} v_{\scriptscriptstyle RT}) \, \partial _{y} v_{\scriptscriptstyle RT} - (x \cdot \nabla _{x} v_{\scriptscriptstyle LT}) \, \partial _{y} v_{\scriptscriptstyle LT} \right] \, \mathrm{d}x. \end{aligned}$$

Then, thanks to (5.1) and the equation satisfied by *v*, we conclude that

$$\begin{aligned}&(n-1) \int _{B_r} |\nabla v|^2 \, \mathrm{d}z = r \int _{\partial B_r} \left[ | \nabla _{\tau } v |^2 - v_{\nu }^2 \right] \, \mathrm{d}{\mathcal {H}}^n + 4 \int _{B^n_r} \partial _{y} v_{\scriptscriptstyle RT} (x \cdot \nabla _{x} v) \, \mathrm{d}x \\&\quad = r \int _{\partial B_r} \left[ | \nabla _{\tau } v |^2 - v_{\nu }^2 \right] \, \mathrm{d}{\mathcal {H}}^n + 4 \int _{B^n_r} ( g' (2 v) - g' (0^+) )(x \cdot \nabla _{x} v) \, \mathrm{d}x. \end{aligned}$$

$$\square $$

We will also need the following lemma:

### Lemma 5.3

For every $$r \in (0, r_0)$$

$$\begin{aligned} \int _{B_r} | \nabla v |^2 \, \mathrm{d}z = \int _{\partial B_r} v \, v_{\nu } \, \mathrm{d}{\mathcal {H}}^n - 2 \int _{B^n_r} v ( g' (2v) - g' (0^+) ) \, \mathrm{d}x. \end{aligned}$$

### Proof

Since *v* is harmonic in $B_r {\setminus } \{ y = 0 \}$,

$$\begin{aligned}&\int _{B_r} \text {div} (v \nabla v) \, \mathrm{d}z = \int _{B_r \cap \{ y> 0\}} \text {div} (v \nabla v) \, \mathrm{d}z + \int _{B_r \cap \{ y< 0\}} \text {div} (v \nabla v) \, \mathrm{d}z\\&\quad = \int _{B_r \cap \{ y> 0\}} | \nabla v |^2 \, \mathrm{d}z + \int _{B_r \cap \{ y< 0\}} | \nabla v |^2 \, \mathrm{d}z \\&\qquad + \int _{B_r \cap \{ y > 0\}} v \Delta v \, \mathrm{d}z + \int _{B_r \cap \{ y < 0\}} v \Delta v \, \mathrm{d}z = \int _{B_r} | \nabla v |^2 \, \mathrm{d}z \end{aligned}$$

On the other hand, applying the divergence theorem in each half-sphere

$$\begin{aligned}&\int _{B_r} \text {div} (v \nabla v) \, \mathrm{d}z = \int _{\partial (B_r \cap \{ y > 0\})} v \, v_{\nu } \, \mathrm{d}{\mathcal {H}}^n + \int _{\partial (B_r \cap \{ y < 0\})} v \, v_{\nu } \, \mathrm{d}{\mathcal {H}}^n\\&\quad = \int _{\partial B_r} v \, v_{\nu } \, \mathrm{d}{\mathcal {H}}^n + \int _{B^n_r} \left[ v_{\scriptscriptstyle LT} \, \partial _y v_{\scriptscriptstyle LT} - v_{\scriptscriptstyle RT} \, \partial _y v_{\scriptscriptstyle RT} \right] \, \mathrm{d}x \\&\quad =\int _{\partial B_r} v \, v_{\nu } \, \mathrm{d} {\mathcal {H}}^n - 2 \int _{B^n_r} v \, \partial _y v_{\scriptscriptstyle RT} \, \mathrm{d}x \\&\quad = \int _{\partial B_r} v \, v_{\nu } \, \mathrm{d} {\mathcal {H}}^n - 2 \int _{B^n_r} v ( g' (2v) - g' (0^+) ) \, \mathrm{d}x, \end{aligned}$$

where we also used (5.1). Comparing the last two chains of equalities we conclude. $\quad \square$

We now start by differentiating $F_v(r)$.

### Lemma 5.4

For every $$r \in (0, r_0)$$

$$\begin{aligned} F_v' (r)&= \int _{\partial B_r} 2 \, v \, v_{\nu } \, \mathrm{d} {\mathcal {H}}^n + \frac{n}{r} F_v (r) \\&= 2 \int _{B_r} | \nabla v |^2 \, \mathrm{d}z + 4 \int _{B^n_r} v ( g' (2v) - g' (0^+) ) \, \mathrm{d}x + \frac{n}{r} F_v (r). \end{aligned}$$

### Proof

Writing the integral in polar coordinates and differentiating we obtain

$$\begin{aligned} F_v' (r)&= \frac{\mathrm{d}}{\mathrm{d}r} \left[ \int _{{\mathbb {S}}^{n}} v^2 (r \omega ) \, r^n \mathrm{d} {\mathcal {H}}^n (\omega ) \right] \\&= \int _{{\mathbb {S}}^{n}} 2 \, v (r \omega ) \nabla v (r \omega ) \cdot \omega \, r^n {\mathcal {H}}^n (\omega ) + n \int _{{\mathbb {S}}^{n}} v^2 (r \omega ) \, r^{n-1} \mathrm{d}{\mathcal {H}}^n (\omega ) \\&= \int _{\partial B_r} 2 \, v v_{\nu } \, \mathrm{d} {\mathcal {H}}^n + \frac{n}{r} \int _{\partial B_r} v^2 (z) \, \mathrm{d} {\mathcal {H}}^n \\&= 2 \int _{B_r} | \nabla v |^2 \, \mathrm{d}z + 4 \int _{B^n_r} v ( g' (2v) - g' (0^+) ) \, \mathrm{d}x + \frac{n}{r} F_v (r), \end{aligned}$$

where the last equality follows by Lemma 5.3. $\quad \square$

We now state a trace inequality, whose proof can be found in [20].

### Lemma 5.5

For any $r > 0$ and any function $w \in W^{1,2} (B_r)$ we have

$$\begin{aligned} \int _{\partial B_r} | w - \overline{w} |^2 \, \mathrm{d} {\mathcal {H}}^n \le C r \int _{B_r} | \nabla w |^2 \, \mathrm{d}z, \end{aligned}$$

where

$$\begin{aligned} \overline{w}:= \frac{1}{\mathcal {H}^{n}(\partial B_{r})} \int _{\partial B_r} w \, \mathrm{d}{\mathcal {H}}^{n}, \end{aligned}$$

and *C* is a constant depending only on the dimension *n*.

In the following we will need an improvement of Lemma 5.5, which can be obtained using the fact that *v* is superharmonic.

### Lemma 5.6

There exists a constant *C*, depending only on *n*, such that for any $r \in (0, r_0)$

$$\begin{aligned} F_v (r) = \int _{\partial B_r} v^2 \, \mathrm{d} {\mathcal {H}}^n \le C r \int _{B_r} | \nabla v |^2 \, \mathrm{d}z, \end{aligned}$$

and

$$\begin{aligned} \int _{B^n_r} v^2 \, \mathrm{d}{\mathcal {H}}^n \le C r \int _{B_r} | \nabla v |^2 \, \mathrm{d}z. \end{aligned}$$

### Proof

Let us start by proving the first inequality. Since $v \in W^{1,2} (B_r)$, by Lemma 5.5

5.6 $$\begin{aligned} F_v (r) = \int _{\partial B_r} v^2 \, \mathrm{d}{\mathcal {H}}^n \le C r \int _{B_r} | \nabla v |^2 \, \mathrm{d}z + \overline{v} \int _{\partial B_r} v \, \mathrm{d}{\mathcal {H}}^n. \end{aligned}$$

Let now ${\widetilde{v}}$ be given by (5.2). Since ${\widetilde{v}}$ is superharmonic,

$$\begin{aligned} 0= & {} {\widetilde{v}} (0) {\mathcal {H}}^n (\partial B_{r}) \ge \overline{{\widetilde{v}}}{\mathcal {H}}^n (\partial B_{r}) = \int _{\partial B_r} {\widetilde{v}} \, \mathrm{d}{\mathcal {H}}^n\\= & {} \int _{\partial B_r} v \, \mathrm{d}{\mathcal {H}}^n = \int _{\partial B_r} v^+ \, \mathrm{d}{\mathcal {H}}^n + \int _{\partial B_r} v^- \, \mathrm{d}{\mathcal {H}}^n. \end{aligned}$$

The above inequality implies that

5.7 $$\begin{aligned} \int _{\partial B_r} v^+ \, \mathrm{d}{\mathcal {H}}^n \le - \int _{\partial B_r} v^- \, \mathrm{d}{\mathcal {H}}^n = \int _{\partial B_r} |v^-| \, \mathrm{d}{\mathcal {H}}^n. \end{aligned}$$

Since $v (x, 0) \ge 0$, we have $v^- (x, 0) = 0$. Thus, by rescaling,

$$\begin{aligned} \frac{r\int _{B_r}|\nabla v^-|^2\mathrm{d}z}{\int _{\partial B_r} (v^-)^2\mathrm{d} {\mathcal {H}}^n}\ge \min \biggl \{ \frac{\int _{B_1}|\nabla w|^2\mathrm{d}z}{\int _{\partial B_1} w^2\mathrm{d} {\mathcal {H}}^n}\,:\,w:B_1\rightarrow \mathbb {R},\,\,w|_{B_1^n}=0\biggr \}=:c_n>0, \end{aligned}$$

where the positivity of $c_n$ follows by a standard compactness argument (actually, by spectral analysis theory, the minimum is attained by $w(x,y)=y$, thus $c_n=1$).

Hence, by Hölder inequality,that combined with (5.7) yields

$$\begin{aligned} \int _{\partial B_r} | v | \, \mathrm{d}{\mathcal {H}}^n \le 2 \int _{\partial B_r} | v^- | \, \mathrm{d}{\mathcal {H}}^n \le C r^{\frac{n+1}{2}} \left( \int _{B_r} | \nabla v |^2 \, \mathrm{d}z \right) ^{1/2}. \end{aligned}$$

Finally, plugging this into (5.6) we get

$$\begin{aligned} F_v (r)&\le C \biggr [r \int _{B_r} | \nabla v |^2 \, \mathrm{d}z + \frac{1}{r^n} \left( \int _{\partial B_r} | v | \, \mathrm{d}{\mathcal {H}}^n \right) ^2 \biggr ] \le C r \int _{B_r} | \nabla v |^2 \, \mathrm{d}z, \end{aligned}$$

which proves the first inequality of the statement.

To show the second inequality, it is enough to observe that

$$\begin{aligned} \min \biggl \{ \frac{\int _{B_1}|\nabla w|^2\mathrm{d}z+\int _{\partial B_1} w^2\mathrm{d}{\mathcal {H}}^n}{\int _{B_1^n} w^2\mathrm{d}{\mathcal {H}}^n}\,:\,w:B_1\rightarrow \mathbb {R}\biggr \}=:c_n'>0, \end{aligned}$$

where again positivity of $c_n'$ follows by a standard compactness argument. By rescaling, this implies that

$$\begin{aligned} \int _{B^n_r} v^2 \, \mathrm{d}{\mathcal {H}}^n \le \frac{1}{c_n'} \biggl (r \int _{B_r} | \nabla v |^2 \, \mathrm{d}z+\int _{\partial B_r} v^2 \, \mathrm{d}{\mathcal {H}}^n\biggr ), \end{aligned}$$

and the result follows by the first inequality of the statement. $\quad \square$

Before proving Proposition 5.1 we need another lemma.

### Lemma 5.7

There exists $\overline{r}_0 \in (0, r_0 )$ and a positive constant $C= C (n)$ such that, whenever $F_v (r) > r^{n+4}$, we have

$$\begin{aligned} \int _{B_r} | \nabla v |^2 \, \mathrm{d}z \le 2 \int _{\partial B_r} v \, v_{\nu } \, \mathrm{d}{\mathcal {H}}^n, \end{aligned}$$

and

$$\begin{aligned} \int _{\partial B_r} v \, v_{\nu } \, \mathrm{d}{\mathcal {H}}^n > C r^{n+3}, \end{aligned}$$

for every $$ 0< r < \overline{r}_0$$.

### Proof

Suppose that $F_v (r) > r^{n+4}$. Then, by Lemma 5.6,

$$\begin{aligned} r^{n+4} < F_v (r) \le C r \int _{B_r} | \nabla v |^2 \, \mathrm{d}z, \end{aligned}$$

which implies

5.8 $$\begin{aligned} \int _{B_r} | \nabla v |^2 \, \mathrm{d}z > \frac{r^{n+3}}{C}. \end{aligned}$$

Thanks to Lemma 5.3 and Lemma 5.6, for *r* sufficiently small we have

5.9 $$\begin{aligned}&\int _{\partial B_r} v \, v_{\nu } \, \mathrm{d}{\mathcal {H}}^n = \int _{B_r} | \nabla v |^2 \, \mathrm{d}z + 2 \int _{B_r^n} v \, ( g' (2v) - g' (0^+) ) \, \mathrm{d}x \nonumber \\&\ge \int _{B_r} | \nabla v |^2 \, \mathrm{d}z - 4 \Vert g '' \Vert _{L^{\infty }} \int _{B_r^n} v^2 \, \mathrm{d}x \nonumber \\&\ge (1 - 4 r C \Vert g '' \Vert _{L^{\infty }} ) \int _{B_r} | \nabla v |^2 \, \mathrm{d}z \ge \frac{1}{2} \int _{B_r} | \nabla v |^2 \, \mathrm{d}z. \end{aligned}$$

This shows the first inequality which, together with (5.8), allows us to conclude.

$$\square $$

We are now ready to prove Proposition 5.1.

### Proof of Proposition 5.1

Since $r\mapsto \max \{ F_v (r) , r^{n+4} \}$ is a semiconvex function (being the maximum between two smooth functions) and $\Phi _v (r) = n+4$ on the region where $\max \{ F_v (r) , r^{n+4} \} = r^{n+4}$, it suffices to prove the monotonicity of $\Phi _v(r)e^{Cr}$ in the open set $\{r\,:\,F_v (r) > r^{n+4}\}$.

Note that, thanks to Lemma 5.4,

5.10 $$\begin{aligned} \Phi _v (r) = r \frac{F'_v (r)}{F_v (r)} = \frac{2 r}{F_v (r)} \int _{\partial B_r} v \, v_{\nu } \, \mathrm{d}{\mathcal {H}}^n + n. \end{aligned}$$

Setting $\Psi _v (r):= \Phi _v (r) - n$, the logarithmic derivative of $\Psi _v$ is given by

5.11 $$\begin{aligned} \frac{\Psi _v' (r)}{\Psi _v (r)}&= \frac{d}{dr} \log \left( \frac{r}{F_v (r)} \int _{\partial B_r} v \, v_{\nu } \, \mathrm{d}{\mathcal {H}}^n \right) \nonumber \\&= \frac{\mathrm{d}}{\mathrm{d}r} \left( \log r + \log \int _{\partial B_r} v \, v_{\nu } \, \mathrm{d}{\mathcal {H}}^n - \log ( F_v (r) ) \right) \nonumber \\&= \frac{1}{r} + \frac{ \displaystyle \frac{d}{d r} \int _{\partial B_r} v \, v_{\nu } \, \mathrm{d}{\mathcal {H}}^n}{\displaystyle \int _{\partial B_r} v \, v_{\nu } \, \mathrm{d}{\mathcal {H}}^n} - \frac{2 \displaystyle \int _{\partial B_r} v \, v_{\nu } \, \mathrm{d}{\mathcal {H}}^n}{F_v (r)} - \frac{n}{r}, \end{aligned}$$

where we used again Lemma 5.4. We now divide the remaining part of the proof into several steps. In the following, it will be convenient to define

$$\begin{aligned} I_1(r):&= 2 \int _{\partial B^n_r} v ( g' (2v) - g' (0^+) ) \, \mathrm{d}{\mathcal {H}}^{n-1} , \\ I_2(r):&= - \frac{2(n-1)}{r} \int _{B_r^n} v ( g' (2v) - g' (0^+) ) \, \mathrm{d}x , \\ I_3(r):&= - \frac{4}{r} \int _{B^n_r} ( g' (2v) - g' (0^+) ) (x \cdot \nabla _{x} v) \, \mathrm{d}x. \end{aligned}$$

**Step 1:** We show that

$$\begin{aligned} \frac{d}{d r} \int _{\partial B_r} v \, v_{\nu } \, \mathrm{d}{\mathcal {H}}^n&= \frac{n-1}{r} \int _{\partial B_r} v \, v_{\nu } \, \mathrm{d}{\mathcal {H}}^n + 2 \int _{\partial B_r} v_{\nu }^2 \, \mathrm{d}{\mathcal {H}}^n\\&\quad + I_1(r) + I_2(r) + I_3(r). \end{aligned}$$

Indeed, thanks to Lemma 5.3,

5.12 $$\begin{aligned}&\frac{d}{d r} \int _{\partial B_r} v \, v_{\nu } \, \mathrm{d}{\mathcal {H}}^n = \frac{d}{d r} \int _{B_r} | \nabla v |^2 \, \mathrm{d}z + 2 \frac{d}{d r} \int _{B_r^n} v ( g' (2v) - g' (0^+) ) \, \mathrm{d}x \nonumber \\&\quad = \int _{\partial B_r} | \nabla v |^2 \, \mathrm{d}{\mathcal {H}}^n + I_1(r). \end{aligned}$$

Using first Lemma 5.2 and then Lemma 5.3, the first integral in the last expression can be written as

$$\begin{aligned}&\int _{\partial B_r} |\nabla v|^2 \, \mathrm{d}{\mathcal {H}}^n = \frac{n-1}{r} \int _{B_r} |\nabla v|^2 \, \mathrm{d}z + 2 \int _{\partial B_r} v_{\nu }^2 \, \mathrm{d}{\mathcal {H}}^n + I_3 (r) \\&\quad = \frac{n-1}{r} \int _{\partial B_r} v \, v_{\nu } \, \mathrm{d}{\mathcal {H}}^n + I_2 (r) + 2 \int _{\partial B_r} v_{\nu }^2 \, \mathrm{d}{\mathcal {H}}^n + I_3 (r). \end{aligned}$$

Inserting this last equality into (5.12), we obtain the claim.

**Step 2:** We prove that

$$\begin{aligned} I_1(r)&+ I_2(r) + I_3(r) \ge - C \int _{B_r} | \nabla v |^2 \, \mathrm{d}z - C r^{ n + \frac{7}{2}}. \end{aligned}$$

Indeed,

$$\begin{aligned} \frac{1}{2} I_3 ( r )&= - \frac{2}{r} \int _{B^n_r } ( g' (2v) - g' (0^+) ) (x \cdot \nabla _{x} v) \, \mathrm{d}x \\&= - \frac{2}{r} \int _{B^n_r } \text {div}_{x} \big ( v ( g' (2v) - g' (0^+) ) x \big ) \, \mathrm{d}x\\&\qquad + \frac{2}{r} \int _{B^n_r } v ( g' (2v) - g' (0^+) ) (\text {div}_{x} x ) \, \mathrm{d}x \\&\qquad + \frac{2}{r} \int _{B^n_r } v \, x \cdot \nabla _{x} ( g' (2v) ) \, \mathrm{d}x \\&= - I_1 ( r ) - I_2 ( r ) + \frac{2}{r} \int _{B^n_r } v ( g' (2v) - g' (0^+) ) \, \mathrm{d}x\\&\qquad + \frac{2}{r} \int _{B^n_r } 2 v (x \cdot \nabla _{x} v) g'' (2v) \, \mathrm{d}x. \end{aligned}$$

Therefore,

5.13 $$\begin{aligned} I_1(r)&+ I_2(r) + I_3(r) \nonumber \\&= \frac{1}{2} I_3 ( r ) + \frac{2}{r} \int _{B^n_r } v ( g' (2v) - g' (0^+) ) \, \mathrm{d}x + \frac{2}{r} \int _{B^n_r } 2 v (x \cdot \nabla _{x} v) g'' (2v) \, \mathrm{d}x. \end{aligned}$$

Let us first estimate the second term in the right hand side of the identity above. Thanks to Lemma 5.6,

5.14 $$\begin{aligned}&\frac{2}{r} \int _{B^n_r } v ( g' (2v) - g' (0^+) ) \, \mathrm{d}x \ge - \frac{4 \Vert g'' \Vert _{L^{\infty }}}{r} \int _{B^n_r } v^2 \, \mathrm{d}x \ge - C \int _{B_r} | \nabla v |^2 \, \mathrm{d}z. \end{aligned}$$

Let us now estimate the remaining two terms. There exists $\tau = \tau (x) \in (0,1)$ such that

5.15 $$\begin{aligned}&\frac{1}{2} I_3 ( r ) + \frac{2}{r} \int _{B^n_r } 2 v (x \cdot \nabla _{x} v) g'' (2v) \, \mathrm{d}x \nonumber \\&\quad = \frac{2}{r} \int _{B^n_r } (x \cdot \nabla _{x} v) \Big ( 2 v g'' (2v) - g' (2v) + g' (0^+) \Big ) \, \mathrm{d}x \nonumber \\&\quad = \frac{2}{r} \int _{B^n_r } 2 v (x \cdot \nabla _{x} v) ( g'' (2v) - g'' (2 \tau v) ) \, \mathrm{d}x \nonumber \\&\quad \ge -\frac{8 \Vert g''' \Vert _{L^{\infty }}}{r} \int _{B^n_r } v^{2} |x \cdot \nabla _{x} v | \, \mathrm{d}x \nonumber \\&\quad \ge - 8 C \Vert g''' \Vert _{L^{\infty }} \, r^{3 + \frac{1}{2}} \int _{B^n_r }\, \mathrm{d}x \ge - C r^{ n + \frac{7}{2}}, \end{aligned}$$

where we used that, by the optimal regularity of *v* (see Theorem 1.1), $|v|\le Cr^{3/2}$ and $|\nabla v|\le Cr^{1/2}$. Combining (5.13)–(5.15), for *r* sufficiently small the claim follows.

**Step 3:** We conclude. Recalling that $\Psi _v (r)= \Phi _v (r) - n$, from Step 1 we have

$$\begin{aligned} \frac{\Psi _v'(r)}{\Psi _v(r)}&= \frac{1}{r} - \frac{n}{r} + \frac{ \displaystyle \frac{d}{d r} \int _{\partial B_r} v \, v_{\nu } \, \mathrm{d}{\mathcal {H}}^n}{\displaystyle \int _{\partial B_r} v \, v_{\nu } \, \mathrm{d}{\mathcal {H}}^n} - \frac{2 \displaystyle \int _{\partial B_r} v \, v_{\nu } \, \mathrm{d}{\mathcal {H}}^n}{F_u (r)} \\&= \frac{\displaystyle 2 \int _{\partial B_r} v_{\nu }^2 \, \mathrm{d}{\mathcal {H}}^n}{\displaystyle \int _{\partial B_r} v \, v_{\nu } \, \mathrm{d} {\mathcal {H}}^n} - \frac{\displaystyle 2 \int _{\partial B_r} v \, v_{\nu } \, \mathrm{d} {\mathcal {H}}^n}{\displaystyle \int _{\partial B_r} v^2 \, \mathrm{d} {\mathcal {H}}^n} + \frac{I_1(r) + I_2(r) + I_3(r)}{\displaystyle \int _{\partial B_r} v \, v_{\nu } \, \mathrm{d}{\mathcal {H}}^n}\\&\ge \frac{I_1(r) + I_2(r) + I_3(r)}{\displaystyle \int _{\partial B_r} v \, v_{\nu } \, \mathrm{d}{\mathcal {H}}^n}, \end{aligned}$$

where in the last step we used Hölder inequality and the fact that, by Lemma 5.7, the integral at the denominator is positive. Then, thanks to Step 2 and Lemma 5.7 again, we obtain

$$\begin{aligned} \frac{\Psi _v'(r)}{\Psi _v(r)}&\ge - C \frac{\displaystyle \int _{B_r} | \nabla v |^2 \, \mathrm{d}z}{\displaystyle \int _{\partial B_r} v \, v_{\nu } \, \mathrm{d}{\mathcal {H}}^n} - C \frac{ r^{ n + \frac{7}{2} } }{\displaystyle \int _{\partial B_r} v \, v_{\nu } \, \mathrm{d}{\mathcal {H}}^n} \\&\ge - C - C r^{n + \frac{7}{2} - (n+3)} \ge - C. \end{aligned}$$

The previous chain of inequalities gives

$$\begin{aligned} 0 \le \left[ \log \Psi _v (r) + C r \right] ' = \left[ \log \Big ( \Psi _v (r) e^{C r} \Big ) \right] '. \end{aligned}$$

Recalling that $\Psi _v (r) = \Phi _v (r) - n$, this shows that $r \mapsto (\Phi _v (r) - n) e^{C r}$ is increasing, and thus the conclusion. $\quad \square$

## Blow Up Profiles and Regularity of the Free Boundary

We are now going to study the blow up profiles of *v* and the regularity of the free boundary. As in the previous section, with no loss of generality we will assume that (0, 0) is a free boundary point and that $v (x, 0) \ge 0$ for every $x \in B^n_{\overline{r}_0}$, where $\overline{r}_0$ is given by Proposition 5.1.

We define, for every $r \in (0,\overline{r}_0)$, the function $v_r : B_1 \rightarrow {\mathbb {R}}$ as

6.1 $$\begin{aligned} v_r (z) := \frac{v (r z)}{d_r} , \qquad d_r:= \left( \frac{F_v (r)}{r^n} \right) ^{\frac{1}{2}}, \end{aligned}$$

where $F_v$ is as in Proposition 5.1. Note that

6.2 $$\begin{aligned} \int _{\partial B_1} v_r^2 \, \mathrm{d}{\mathcal {H}}^n = 1 \qquad \text { for every } r < \overline{r}_0. \end{aligned}$$

The next proposition shows which are the possible values of $\Phi _v (0^+)$.

### Proposition 6.1

Let the assumptions of Theorem 1.1 be satisfied, suppose that (0, 0) is a free boundary point for *u*, and that $u(x,0)\ge 0$ in $B_{{\overline{r}}_0}^n$. Let *v* be given by (1.5), and let $\Phi _v$ be as in Proposition 5.1. Then, either $\Phi _v (0^+) = n+3$, or $\Phi _v (0^+) \ge n+4$.

We first prove the proposition above in the case

$$\begin{aligned} \liminf _{r \rightarrow 0^+} \frac{d_r}{r^2} < + \infty . \end{aligned}$$

### Proof of Proposition 6.1

*when*$$\liminf _{r\rightarrow 0^+} d_r/r^2 < + \infty $$.

We will show that in this case we always have $\Phi _v (0^+) \ge n+4$. Indeed, by assumption there exists $C > 0$ such that

$$\begin{aligned} \frac{d_r^2}{r^4} = \frac{F_v (r)}{r^{n+4}} \le C \qquad \text { for every } r \in (0, 1). \end{aligned}$$

We then have two possibilities (see also the second part of the proof of [9, Lemma 6.1]).

**Case 1:** there exists a sequence $(r_j)_{j \in {\mathbb {N}}}$ with $r_j \rightarrow 0$ such that

$$\begin{aligned} F_v (r_j) < r_j^{n+4} \qquad \text {for}~j~\hbox {sufficiently large}. \end{aligned}$$

Then, $\Phi _v (r_j)= n+4$ for *j* sufficiently large, and therefore $\Phi _v (0^+) = n+4$.

**Case 2:** for *r* sufficiently small

$$\begin{aligned} r^{n+4} \le F_v (r) \le C r^{n+4}. \end{aligned}$$

Then,

6.3 $$\begin{aligned} ( n+4 ) \log r \le \log F_v (r) \le \log C + ( n+4 ) \log r. \end{aligned}$$

Suppose now, by contradiction, that there exists $\eta > 0$ such that

$$\begin{aligned} \Phi _v (r) \le n+4 - \eta \quad \text { for}~ r~ \text { sufficiently small}, \end{aligned}$$

and let $(r_j)_{j \in {\mathbb {N}}}$ be a strictly decreasing sequence with $r_j \rightarrow 0$. Then, thanks to (6.3), for every $k, l \in {\mathbb {N}}$ with $k < l$ we have

$$\begin{aligned} ( n+4 ) \log r_k \le \log F_v (r_k) \quad \text { and } \quad \log F_v (r_l) \le \log C + ( n+4 ) \log r_l. \end{aligned}$$

Therefore, by the definition of $\Phi _v$,

$$\begin{aligned}&( n+4 ) ( \log r_k - \log r_l) - \log C \le \log F_v (r_k) - \log F_v (r_l) = \int _{r_l}^{r_k} \frac{d}{dr} \log F_v (r) \, dr \\&= \int _{r_l}^{r_k} \frac{\Phi _v (r)}{r} \, dr \le (n+4 - \eta ) ( \log r_k - \log r_l), \end{aligned}$$

which is impossible if we choose $\log r_k - \log r_l \rightarrow \infty$. $\quad \square$

In the next proposition we consider the case

$$\begin{aligned} \liminf _{r \rightarrow 0^+} \frac{d_r}{r^2} = + \infty . \end{aligned}$$

### Proposition 6.2

Let the assumptions of Theorem 1.1 be satisfied, suppose that (0, 0) is a free boundary point for *u*, and that $u(x,0)\ge 0$ in $B_{{\overline{r}}_0}^n$. Let *v* be given by (1.5), and let $F_v$ and $\Phi _v$ be as in Proposition 5.1. Define $v_r$ as in (6.1), and assume that

$$\begin{aligned} \liminf _{r \rightarrow 0^+} \frac{d_r}{r^2} = + \infty . \end{aligned}$$

Then, there exists a sequence $(r_k)_{k \in {\mathbb {N}}}$ with $r_k \rightarrow 0$, and a homogeneous function $v_{\infty } \in W^{1,2} (B_1)$ with homogeneity degree $1/2(\Phi _v (0^+) - n)$, such that

$$\begin{aligned} v_{r_k} \rightharpoonup v_{\infty } \quad \text { weakly in } W^{1,2} (B_1), \end{aligned}$$

and

6.4 $$\begin{aligned} v_{r_k} \rightarrow v_{\infty } \text { in}~ C^{1, \gamma }~ \text {on compact subsets of } B_{1} \cap \{ y \ge 0 \}, \end{aligned}$$

for any $\gamma \in (0, 1/2)$. Moreover, $v_{\infty }$ satisfies the classical Signorini problem in $B_1$ and is even with respect to *y*:

6.5 $$\begin{aligned} {\left\{ \begin{array}{ll} \Delta v_{\infty } = 0 &{}\quad \text { in } B_1 {\setminus } \{ y = 0 \}, \\ v_{\infty } \ge 0 &{}\quad \text { on } B^n_1, \\ \partial _y v_{\infty } \le 0 &{}\quad \text { on } B^n_1, \\ v_{\infty } \partial _y v_{\infty } = 0 &{}\quad \text { on } B^n_1, \\ v_{\infty } (x, - y) = v_{\infty } (x, y) &{}\quad \text { in } B_1. \\ \end{array}\right. } \end{aligned}$$

Finally, it holds that $\Phi _v (0^+) = n + 3$ and that, up to a multiplicative constant and to a change of variables, we have

6.6 $$\begin{aligned} v_{\infty } (x, y) = \rho ^{3/2} \cos \frac{3}{2} \theta , \end{aligned}$$

where $$\rho ^2 = x_n^2 + y^2$$ and $$\tan \theta = y/x_{n}$$.

### Proof of Proposition 6.2

*and conclusion of proof of Proposition* 6.1.

Since $d_r/r^2 \rightarrow \infty$, for *r* sufficiently small we have $F_v (r) > r^{n+4}$. Then, thanks to Proposition 5.1 and by (5.10), for $r < \overline{r}_0$ we have

$$\begin{aligned} \Phi _v (\overline{r}_0) e^{C (\overline{r}_0 - r)} \ge \Phi _v (r) = 2 r \frac{\displaystyle \int _{\partial B_r} v \, v_{\nu } \, \mathrm{d}{\mathcal {H}}^n}{\displaystyle \int _{\partial B_r} v^2 \, \mathrm{d} {\mathcal {H}}^n} + n. \end{aligned}$$

Therefore, by (5.9) and the definition of $v_r$,

$$\begin{aligned} \Phi _v (\overline{r}_0) e^{C (\overline{r}_0- r)} - n \ge 2 r \,\frac{ \displaystyle \int _{\partial B_r} v \, v_{\nu } \, \mathrm{d} {\mathcal {H}}^n}{\displaystyle \int _{\partial B_r} v^2 \, \mathrm{d} {\mathcal {H}}^n} = 2 r \,\frac{\frac{1}{2} \displaystyle \int _{B_r} | \nabla v |^2 \, \mathrm{d}z }{\displaystyle \int _{\partial B_r} v^2 \, \mathrm{d} {\mathcal {H}}^n} =\int _{B_1} | \nabla v_r |^2 \, \mathrm{d}z, \end{aligned}$$

for *r* sufficiently small. Consider now a sequence $r_k \rightarrow 0$. By the previous inequality and thanks to (6.2), the sequence $(v_{r_k})_{k \in {\mathbb {N}}}$ is bounded in $W^{1,2} (B_1)$. Thus, up to subsequences,

$$\begin{aligned} v_{r_k} \rightharpoonup v_{\infty } \text { weakly in } W^{1,2} (B_1), \end{aligned}$$

for some $v_{\infty } \in W^{1,2} (B_1)$. Thanks to the uniform $C^{1,1/2}$ regularity for solutions, we also have that (6.4) holds. Let us show that $v_{\infty } \partial _y v_{\infty } = 0$ on $B^n_1$, since the other conditions in (6.5) are a direct consequence of (6.4). Recalling the definition of $v_{r_k}$, from the identity

$$\begin{aligned} v (rx, 0) \left[ \partial _y v (rx, 0) - g' (2 v (rx, 0)) + g' (0^+) \right] = 0 \quad \text { for every } x \in B_1^n \end{aligned}$$

it follows that, for every $k \in {\mathbb {N}}$,

6.7 $$\begin{aligned} v_{r_k} (x, 0) \left[ \partial _y v_{r_k} (x, 0) - \frac{r_k}{d_{r_k}} ( g' ( 2 d_{r_k} v_{r_k} (x, 0)) - g' (0^+) ) \right] =0. \end{aligned}$$

Thanks to (6.4), since

$$\begin{aligned} \frac{r_k}{d_{r_k}} | g' ( 2 d_{r_k} v_{r_k} (x, 0) ) - g' (0^+) | \le 2 r_k v_{r_k} (x, 0) \Vert g'' \Vert _{L^{\infty }} {\mathop {\rightarrow }\limits ^{k\rightarrow \infty }} 0 \qquad \text { for every } x \in B_1^n, \end{aligned}$$

taking the limit in (6.7) we obtain

$$\begin{aligned} v_{\infty } \partial _y v_{\infty } = 0 \qquad \text { in } B^n_1. \end{aligned}$$

To show that $v_\infty$ is homogeneous, let us first prove that $\Phi _{v_{\infty }}$ is constant for *r* sufficiently small. Indeed, let $r < s \ll 1$. A direct calculation shows that

$$\begin{aligned} \Phi _{v_{r_k}} (r) - \Phi _{v_{r_k}} (s) = \Phi _v (r_k r) - \Phi _v (r_k s) \qquad \text { for every } k \in {\mathbb {N}}. \end{aligned}$$

Thanks to (6.4), taking the limit as $k \rightarrow \infty$ we obtain

$$\begin{aligned} \Phi _{v_{\infty }} (r) - \Phi _{v_{\infty }} (s) = 0, \end{aligned}$$

where we used the existence of the limit $\lim _{r\rightarrow 0^+}\Phi _v (r)$, which follows from Proposition 5.1. Since $v_{\infty }$ satisfies the Signorini problem (6.5), from [6, Lemma 1] it follows that $v_{\infty }$ is homogeneous and that

$$\begin{aligned} \Phi _v (0^+) = 2 \mu + n, \end{aligned}$$

where $\mu$ is the homogeneity degree of $v_{\infty }$. Therefore,

$$\begin{aligned} \mu = \frac{\Phi _v (0^+) - n}{2}. \end{aligned}$$

Arguing as in [9, Lemma 6.6], one gets that $F_v (r) \le C r^{2 \mu + n}$. Since $d_r/r^2\rightarrow \infty$, this implies $\mu <2$, and one concludes as in [6, Section 4] that $\mu =3/2$ and that the function $v_{\infty }$ is given by (6.6). $\quad \square$

## $\varvec{C^{1, \alpha }}$ Regularity of the Free Boundary for $\varvec{\mu =}$ 3/2.

We now study the regularity of the free boundary in the special case in which $\Phi _v (0^+) = n + 3$. Note that, by the argument in the previous section, this corresponds to the case

$$\begin{aligned} \liminf _{r \rightarrow 0^+} \frac{d_r}{r^2} = + \infty . \end{aligned}$$

We start by proving the $C^1$ regularity.

### Lemma 7.1

Let the assumptions of Theorem 1.1 be satisfied, suppose that (0, 0) is a free boundary point for *u*, and that $u(x,0)\ge 0$ in $B_{{\overline{r}}_0}^n$. Let *v* be given by (1.5), and let $\Phi _v$ be as in Proposition 5.1. Assume that $\Phi _v (0^+) = n+3$, and choose a coordinate system in $\mathbb {R}^n$ such that (6.6) holds true. Then, for every $\overline{c} > 0$ there exists ${\overline{\rho }} = {\overline{\rho }} (\overline{c}) > 0$ with the following property: For every $\tau \in {\mathbb {S}}^n \cap \{ y = 0 \}$ with $\tau \cdot {\mathbf {e}}_n \ge \overline{c}$ we have

7.1 $$\begin{aligned} \partial _{\tau } v (z) \ge 0, \qquad \text { for every } z \in B_{{\overline{\rho }}}. \end{aligned}$$

In addition, near the origin the free boundary of *v* is the graph of a $C^1$ function

$$\begin{aligned} x_n = f (x_1, \ldots , x_{n-1}). \end{aligned}$$

Before giving the proof of Lemma 7.1 we make some useful observation on the tangential derivatives of the functions $v_{r_k}$ introduced in the previous section.

Let us fix $\overline{c} > 0$ and ${\mathbf {e}} \in {\mathbb {S}}^n \cap \{ y = 0 \}$ with ${\mathbf {e}} \cdot {\mathbf {e}}_n = 0$. Choose now $a \ge \overline{c}$ and $b \in {\mathbb {R}}$ such that $a^2 + b^2 = 1$, and define $h_k : B_1 :\rightarrow {\mathbb {R}}$ as the sequence of functions given by

7.2 $$\begin{aligned} h_k := \partial _{\tau } v_{r_k} \qquad \text { for every } k \in {\mathbb {N}}, \end{aligned}$$

where $\tau := a {\mathbf {e}}_n + b {\mathbf {e}}$. For any $\eta \in (0, 1/(8n))$, thanks to (6.6) and (6.4), there exist $k_0 = k_0 (a, b, \eta )$ and $c_0 = c_0 (a, b , \eta )$ such that the following properties are satisfied for $k > k_0$:

(i) $$\Delta h_k = 0$$ in $$B_{2/3} \cap \{ |y| > 0\}$$;
(ii) $$h_k \ge 0$$ in $$B_{2/3} \cap \{ |y| > \eta \}$$;
(iii) $$h_k \ge c_0$$ in $$B_{2/3} \cap \left\{ |y| > \frac{1}{8 n} \right\} $$;
(iv) $$h_k \ge - C \eta ^{1/2}$$ in $$B_{2/3}$$,

where property (iv) follows from the optimal regularity and (ii). Let us show that we also have

(v) $$\partial _y h_k \le C \eta ^{1/2}$$ on $$B^n_{2/3} \cap \{ h_k \ne 0 \}$$.

To this aim, first of all observe that $B^n_{2/3} \cap \{ h_k \ne 0 \} \subset B^n_{2/3} \cap \{ v_{r_k} \ne 0 \}$. Indeed, if $x \in B^n_{2/3}$ is such that $v_{r_k} (x, 0) = 0$, then

$$\begin{aligned} h_k (x, 0) = (\partial _{\tau } v_{r_k} ) (x, 0) = \frac{r_k}{d_{r_k}} ( \partial _{\tau } v ) (r_k x , 0) = 0, \end{aligned}$$

by nonnegativity of *v* and optimal regularity.

Let now $x \in B^n_{2/3}$ be such that $h_k (x, 0) \ne 0$. Then we have $v_{r_k} (x, 0) > 0$ and, for *k* sufficiently large,

$$\begin{aligned} (\partial _y h_k ) (x, 0)&= \frac{r_k}{d_{r_k}} \partial _{\tau } \left\{ g' (2 v (r_{k} x,0)) - g' (0^+) \right\} = 2 r_k g'' (2 v (r_{k} x,0)) h_k \le C \eta ^{1/2}, \end{aligned}$$

where we used (iv). We now consider a version of [6, Lemma 5] which is useful for our purposes.

### Lemma 7.2

Let $0< \eta < 1/(8n)$, let $C, c_0 > 0$, and let $\sigma : [0, 1] \rightarrow [0, \infty )$ be a continuous function with $\sigma (0)=0$. Suppose that $h : B_1 :\rightarrow {\mathbb {R}}$ satisfies the following assumptions:

(i) $$\Delta h = 0$$ in $$B_1 \cap \{ |y| > 0\}$$;
(ii) $$h \ge 0$$ in $$B_1 \cap \{ |y| > \eta \}$$;
(iii) $$h \ge c_0$$ in $$B_1 \cap \left\{ |y| > \frac{1}{8 n} \right\} $$;
(iv) $$h \ge - \sigma (\eta )$$ in $$B_1$$,
(v) $$\partial _y h \le \sigma (\eta )$$ on $$B^n_1 \cap \{ h \ne 0 \}$$.

Then, there exists $\eta _0 = \eta _0 (n, c_0, \sigma )$ such that if $\eta < \eta _0$ we have $h \ge 0$ in $B_{1/2}$.

### Proof

Suppose, by contradiction, that there exists $\overline{z} = (\overline{x}, \overline{y}) \in B_{1/2}$ such that $h (\overline{z}) < 0$ (note that, by (iii), this implies $\overline{y} < 1/(8n)$). We define

$$\begin{aligned} Q:= \left\{ (x, y) \in {\mathbb {R}}^{n+1} : |x - \overline{x}|< \frac{1}{3}, \, \, 0< | y | < \frac{1}{4 n} \right\} , \end{aligned}$$

and

$$\begin{aligned} P (x, y) : = |x - \overline{x}|^2 - n y^2, \end{aligned}$$

and we set

$$\begin{aligned} w (z) := h (z) + \delta P (z) - \sigma (\eta ) y, \end{aligned}$$

where $\delta > 0$ will be chosen later. Note that *w* is harmonic in *Q* and

$$\begin{aligned} w (\overline{z}) = h (\overline{z}) - \delta \overline{y}^2 - \sigma (\eta ) \overline{y} < 0. \end{aligned}$$

Therefore, there exists a minimum point ${\hat{z}} = ({\hat{x}}, {\hat{y}}) \in \partial Q$ such that

$$\begin{aligned} \min _{z \in \overline{Q}} w (z) = w ({\hat{z}}) < 0. \end{aligned}$$

We have the following possibilities:

**Case 1.**${\hat{z}} \in \partial Q \cap \{ y > 1/(8n)\}$. Thanks to (iii), for $\eta$ and $\delta$ sufficiently small we have

$$\begin{aligned} w ({\hat{z}}) \ge c_0 - \frac{\delta }{16 n} - \frac{\sigma (\eta )}{4 n} > 0, \end{aligned}$$

which is impossible.

**Case 2.**${\hat{z}} \in \partial Q \cap \{ \eta \le y < 1/(8n)\}$. Using property (ii) we obtain that for $\eta$ sufficiently small

$$\begin{aligned} w ({\hat{z}}) \ge \delta \left( \frac{1}{9} - \frac{1}{64 n} \right) - \frac{\sigma (\eta )}{8 n} > 0, \end{aligned}$$

which is impossible.

**Case 3.**${\hat{z}} \in \partial Q \cap \{ (x, y) \in {\mathbb {R}}^{n+1}: |x - \overline{x} | = \frac{1}{3}, 0< y < \eta \}$. Thanks to property (iv), for $\eta$ sufficiently small

$$\begin{aligned} w ({\hat{z}}) \ge - \sigma (\eta ) + \delta \left( \frac{1}{9} - n \eta ^2 \right) - \eta \, \sigma (\eta ) = \delta \left( \frac{1}{9} - n \eta ^2 \right) - (1 + \eta ) \, \sigma (\eta ) > 0, \end{aligned}$$

which is impossible.

**Case 4.**${\hat{z}} \in \partial Q \cap \{ y = 0 \}$. In this case, if ${\hat{z}} \in \{ h = 0 \}$ we obtain

$$\begin{aligned} w ({\hat{z}}) = \delta P ({\hat{z}}) = \delta | {\hat{x}} - \overline{x} |^2 \ge 0, \end{aligned}$$

which is impossible. On the other hand, if ${\hat{z}} \in \{ h \ne 0 \}$, using Hopf Lemma and property (v)

$$\begin{aligned} 0 < \partial _y w ({\hat{z}}) = \partial _y h ({\hat{z}}) - \sigma (\eta ) \le 0, \end{aligned}$$

which is impossible. $\quad \square$

We are now ready to prove that the free boundary is $C^1$.

### Proof of Lemma 7.1

Applying Lemma 7.2 to the functions $h_k$ introduced in (7.2) we obtain (7.1). As a consequence, for every $L > 0$ there exists ${\widetilde{r}} = {\widetilde{r}} (L) > 0$ such that (recall that $K_u$ is defined in (1.3))

$$\begin{aligned} \partial K_u \cap B^n_{{\widetilde{r}}} = \{ (x_1, \ldots , x_n) \in B^n_{{\widetilde{r}}} : x_n = f_L (x_1, \ldots , x_{n-1}) \} \end{aligned}$$

for a suitable Lipschitz continuous function $f_L$ with Lipschitz constant *L*.

Consider now a point ${{\hat{x}}} \in \partial K_u \cap B^n_{{\widetilde{r}}}$ and define the function $v_{{{\hat{x}}}}(x,y):=v(x-{{\hat{x}}},y)$. Note that we can repeat the same argument (frequency formula and blow-up procedure) with $v_{{{\hat{x}}}}$ in place of *v*. Also, observe that since the function ${{\hat{x}}} \mapsto \Phi _{v_{{{\hat{x}}}}}(r)e^{Cr}$ is continuous for $r>0$ fixed, the function ${{\hat{x}}} \mapsto \Phi _{v_{{{\hat{x}}}}}(0^+)$ is upper-semicontinuous (being the infimum over $r \in (0,\overline{r}_0)$ of continuous functions, cf. Proposition 5.1). Hence, since $\Phi _{v_{{{\hat{x}}}}}(0^+) \in \{n+3\}\cup [n+4,\infty )$ (by Proposition 6.1) and by assumption $\Phi _{v_{0}}(0^+)=\Phi _{v}(0^+) = n+3$, we deduce that there exists ${{\hat{r}}}>0$ such that $\Phi _{v_{{{\hat{x}}}}}(0^+)=n+3$ for all ${{\hat{x}}} \in \partial K_u \cap B^n_{{\hat{r}}}$.

This implies that the previous argument can be repeated at every point in $\partial K_u \cap B^n_{{\hat{r}}}$, and it follows that for any $L>0$ there exists ${\widetilde{r}} (L) > 0$ such that $\partial K_u \cap B^n_{{\widetilde{r}} (L)}({{\hat{x}}})$ has Lipschitz constant *L* for any point ${{\hat{x}}} \in \partial K_u \cap B^n_{{\hat{r}}}$. Since $L > 0$ can be made arbitrarily small and the radius ${\widetilde{r}} (L) > 0$ is independent of ${{\hat{x}}}$, this implies that the free boundary is $C^1$ in a neighborhood of the origin. $\quad \square$

### Lemma 7.3

Let the assumptions of Theorem 1.1 be satisfied, suppose that (0, 0) is a free boundary point for *u*, and that $u(x,0)\ge 0$ in $B_{{\overline{r}}_0}^n$. Let *v* be given by (1.5), and let $\Phi _v$ be as in Proposition 5.1. Assume that $\Phi _v (0^+) = n+3$. Then the free boundary is $C^{1, \alpha }$ near (0, 0), for some $\alpha \in (0, 1)$.

### Proof

We start by observing that the function $h (x, y) := \partial _{x_n} v (x, y)$ satisfies

$$\begin{aligned} \partial _y h (x, 0) = 2 g'' (2 v (x,0)) h (x, 0) \qquad \text { if } v (x, 0) > 0. \end{aligned}$$

Therefore, by [10],

7.3 $$\begin{aligned} ( \Delta ^{\frac{1}{2}} h (\cdot , 0) ) (x) = 2 g'' (2 v (x,0)) h (x, 0) + \partial _y \overline{h} (x, 0) - \partial _y h (x, 0) \qquad \text { if } v (x, 0) > 0,\nonumber \\ \end{aligned}$$

where $\overline{h}$ is the harmonic extension of $h (\cdot , 0)$ to ${\mathbb {R}}^n \times (0, \infty )$. Note that $\overline{h} - h$ is smooth near $\{ y = 0 \}$, since it is harmonic in ${\mathbb {R}}^n \times (0, A)$ with zero boundary condition on $\{ y = 0 \}$. For every $0 < r \ll 1$, set

$$\begin{aligned} h_{r} (x) : = \frac{r}{d_r} h (rx, 0), \qquad x \in B^n_1, \end{aligned}$$

where $d_r$ is given by (6.1). From (7.3) it follows that, if $v (rx, 0) > 0$,

$$\begin{aligned}&( \Delta ^{\frac{1}{2}} h_{r} (\cdot , 0)) (x) = \frac{r^2}{d_r} ( \Delta ^{\frac{1}{2}} h (\cdot , 0) ) (r x) \\&= \frac{r^2}{d_r} \left[ 2 g'' (2 v ( r x,0)) h (r x , 0) + \partial _y \overline{h} (r x, 0) - \partial _y h (r x, 0) \right] = : F (x). \end{aligned}$$

Since *v* and *h* are bounded, we have

$$\begin{aligned} | F | \le C\frac{r^2}{d_r} (1+\Vert g'' \Vert _{L^{\infty }}), \end{aligned}$$

for some positive constant *C*. Note also that, for *r* sufficiently small, $h_r \ge 0$ in $B^n_1$ thanks to (7.1). Therefore, using the fact that $h_r = h_r^+$ in $B^n_1$ we obtain

$$\begin{aligned} ( \Delta ^{\frac{1}{2}} h^+_{r} (\cdot , 0)) (x) \ge ( \Delta ^{\frac{1}{2}} h_{r} (\cdot , 0)) (x) = F (x) \quad \text { if } v (rx, 0) > 0. \end{aligned}$$

Moreover, by definition of $h_r$ we have $h_r (x, 0)= (\partial _{e_n} v_r ) (x, 0)$, where $v_r$ is defined in (6.1). Therefore, thanks to (6.4),

$$\begin{aligned} h_r \rightarrow \partial _{x_n} v_{\infty } \quad \text { uniformly in } B^n_{2/3}, \end{aligned}$$

as $r\rightarrow 0^+$. Recalling (6.6), it follows that for *r* small enough

$$\begin{aligned} \sup _{B^n_{1/2}} h^+_r = \sup _{B^n_{1/2}} h_r \ge 1. \end{aligned}$$

Let now $i \in \{ 1, \ldots , n-1\}$, $\tau _i := \frac{{\mathbf {e}}_n + {\mathbf {e}}_i}{\sqrt{2}}$. We can repeat the same argument used for *h* for the function $h_{i} (x, y):= \partial _{\tau _i} v (x, y)$, obtaining that the function $x \mapsto h^+_{i, r} (x, 0) := (r/d_r) h^+_i (rx, 0)$ satisfies

$$\begin{aligned} {\left\{ \begin{array}{ll} ( \Delta ^{\frac{1}{2}} h^+_{i, r} (\cdot , 0)) (x) \ge F_i (x) &{} \text { for every } x \in B^n_1 \text { with } v (rx, 0) > 0, \\ h^+_{i, r} (x, 0) = 0 &{} \text { for every } x \in B^n_1 \text { with } v (rx, 0) = 0, \end{array}\right. } \end{aligned}$$

with

$$\begin{aligned} | F_i | \le C\frac{r^2}{d_r} (1+\Vert g'' \Vert _{L^{\infty }}), \qquad \sup _{B^n_{1/2}} h^+_{i,r} \ge 1. \end{aligned}$$

Since $r^2/d_r \rightarrow 0$, for *r* sufficiently small we can apply [23, Theorem 1.6] to the nonnegative functions $h^+_{r} (\cdot , 0)$ and $h^+_{i,r} (\cdot , 0)$. We then obtain that the ratio $h^+_{i, r}/h^+_r$ is $C^{0, \alpha }$ in $B^n_{1/2}$, for some $\alpha \in (0, 1)$. Since equalities $h^+_{i, r} = h_{i,r}$ and $h^+_{r} = h_{r}$ hold true in $B^n_{1/2}$, it follows that $h_i /h$ is of class $C^{0, \alpha }$ is a neighborhood of the origin. Let now *f* be the function given by Lemma 7.1. Since

$$\begin{aligned} \frac{h_i}{h} = \frac{1}{\sqrt{2}} + \frac{1}{\sqrt{2}} \, \frac{ \partial _{x_i} v (x, 0)}{\partial _{x_n} v (x, 0)}, \end{aligned}$$

and

$$\begin{aligned} \nabla f = - \left( \frac{ \partial _{x_1} v }{\partial _{x_n} v } , \ldots , \frac{ \partial _{x_{n-1}} v}{\partial _{x_n} v} \right) , \end{aligned}$$

this implies that *f* is $C^{1, \alpha }$ in a neighborhood of the origin. $\quad \square$
